# A spliceosome intermediate with loosely associated tri-snRNP accumulates in the absence of Prp28 ATPase activity

**DOI:** 10.1038/ncomms11997

**Published:** 2016-07-05

**Authors:** Carsten Boesler, Norbert Rigo, Maria M. Anokhina, Marcel J. Tauchert, Dmitry E. Agafonov, Berthold Kastner, Henning Urlaub, Ralf Ficner, Cindy L. Will, Reinhard Lührmann

**Affiliations:** 1 Department of Cellular Biochemistry, MPI for Biophysical Chemistry, Am Fassberg 11, D-37077 Göttingen, Germany; 2 Department of Molecular Structural Biology, Institute for Microbiology and Genetics, GZMB, Georg-August-Universität Göttingen, Justus-von-Liebig-Weg 11, D-37077 Göttingen, Germany; 3 Bioanalytical Mass Spectrometry Group, MPI for Biophysical Chemistry, Am Fassberg 11, D-37077 Göttingen, Germany; 4 Bioanalytics Group, Institute for Clinical Chemistry, University Medical Center Göttingen, Robert-Koch-Straße 40, D-37075 Göttingen, Germany

## Abstract

The precise role of the spliceosomal DEAD-box protein Prp28 in higher eukaryotes remains unclear. We show that stable tri-snRNP association during pre-catalytic spliceosomal B complex formation is blocked by a dominant-negative hPrp28 mutant lacking ATPase activity. Complexes formed in the presence of ATPase-deficient hPrp28 represent a novel assembly intermediate, the pre-B complex, that contains U1, U2 and loosely associated tri-snRNP and is stalled before disruption of the U1/5′ss base pairing interaction, consistent with a role for hPrp28 in the latter. Pre-B and B complexes differ structurally, indicating that stable tri-snRNP integration is accompanied by substantial rearrangements in the spliceosome. Disruption of the U1/5′ss interaction alone is not sufficient to bypass the block by ATPase-deficient hPrp28, suggesting hPrp28 has an additional function at this stage of splicing. Our data provide new insights into the function of Prp28 in higher eukaryotes, and the requirements for stable tri-snRNP binding during B complex formation.

## Abstract (web summary)

The assembly of the splicesome involves several distinct stages that require the sequential action of DExD/H-box RNA helicases. Here, the authors uncover a new intermediate, the pre-B complex, that accumulates in the presence of an inactive form of the DEAD-box protein Prp28.

## Graphical abstract

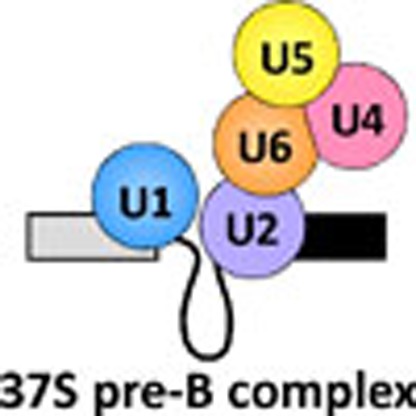

The spliceosome is a highly dynamic molecular machine that assembles *de novo* for each round of splicing by the ordered interaction of five small nuclear ribonucleoproteins (snRNPs) and numerous splicing factors with the pre-mRNA^1^. Initial spliceosome assembly can occur across an exon if the adjacent intron is longer than ∼250 nt. In this case, U2 snRNP binds the branch point sequence (BPS) upstream of the exon and U1 snRNP interacts with the 5′ splice site (ss) downstream of it, forming a cross-exon complex^2^. In contrast, stepwise assembly of the spliceosome across an intron is initiated by interaction of U1 snRNP with the 5′ss and U2 snRNP with the downstream BPS of the intron, yielding the spliceosomal A complex. Stable U4/U6.U5 tri-snRNP binding then generates the pre-catalytic B complex. Subsequently, the B^act^ and then B* complex, which catalyses step I of splicing, are generated. B* is transformed into complex C, which contains the cleaved 5′ exon and the intron-lariat-3′exon splicing intermediates. Complex C then catalyses step II of splicing, during which the lariat intron is excised and the 5′ and 3′ exons are ligated.

The spliceosome undergoes multiple structural and compositional rearrangements, including extensive changes in its RNA–RNA network^3^,^4^. In complex A, the U1 and U2 snRNAs base pair with the 5′ss and BPS, respectively. The U4/U6.U5 tri-snRNP, in which the U4 and U6 snRNAs are extensively base paired, associates with complex A, and a short helix between the 3′end of U6 and 5′end of U2 (U2/U6 helix II) is formed. Subsequently, the U1/5′ss interaction is disrupted, allowing base pairing of the 5′ss with the conserved ACAGA box of U6 snRNA. On unwinding of the U4/U6 duplex, U6 forms a functionally important intramolecular stem-loop and interacts with U2 to form U2/U6 helix I, generating the spliceosome's catalytically active RNA network^5^.

Structural rearrangements in the spliceosome are driven by eight conserved DExD/H-box ATPases/helicases^6^,^7^ that couple the energy of ATP hydrolysis to structural and/or compositional rearrangements at distinct steps of the splicing cycle^8^,^9^. One of these essential factors is the DEAD-box protein Prp28, an integral component of the U5 snRNP and U4/U6.U5 tri-snRNP^10^ in human, but not in *Saccharomyces cerevisiae* (*S. cerevisiae*)^11^. Prp28 contains all eight conserved motifs (I–VIII) present in DEAD-box proteins that are required for ATPase and RNA unwinding activity^10^. In yeast, mutation of these motifs indicated that Prp28′s putative ATPase and helicase activities are required for pre-mRNA splicing *in vivo*^12^. However, isolated Prp28 exhibits very little^13^ or no^11^,^14^,^15^,^16^ ATPase activity *in vitro*, and to date no RNA unwinding activity could be demonstrated. Indeed, isolated yeast and human (h)Prp28 adopt a conformation not conducive for ATP hydrolysis, with their RecA domains in an open conformation^13^,^16^. In human, hPrp28 first binds ATP after incorporation into the spliceosome, suggesting that interactions with other spliceosome components lead to a structural change in hPrp28 that enables ATP binding and potentially triggers its ATPase activity^16^.

In *S. cerevisiae*, Prp28 plays an essential role in destabilizing the U1/5′ss duplex, thereby allowing the exchange of U1 for U6 snRNA interaction with the 5′ss (refs 17, 18). Mutations in yeast U1 snRNP proteins that destabilize the U1/5′ss interaction can bypass the need for Prp28 (refs 18, 19). In human, immunodepletion of hPrp28 from HeLa nuclear extract blocks the A to B complex transition^20^. In addition, phosphorylation of hPrp28 by serine–arginine protein kinase 2 is necessary for hPrp28 integration into the U4/U6.U5 tri-snRNP and only tri-snRNPs containing hPrp28 are competent for stable B complex formation^20^. However, the precise role of Prp28 in higher eukaryotes, including whether its enzymatic activity is required for stable B complex formation, remains unclear.

Stable U4/U6.U5 tri-snRNP integration during human B complex formation not only depends on the presence of hPrp28, but also is aided by other spliceosomal proteins including serine–arginine rich (SR) proteins^21^, the tri-snRNP proteins hSAD1 and hSART1 (ref. 22), and SPF30 (refs 23, 24). During B complex formation, B-specific proteins interact with the spliceosome but are released already during the subsequent activation stage^25^,^26^. These proteins thus potentially aid in stable tri-snRNP integration during B complex formation^25^,^27^. Several tri-snRNP components, including the U5 and U6 snRNAs^28^,^29^ and the proteins Prp8 and Prp28 (refs 30, 31), interact with the 5′ss in mammalian B complexes, and thus likely also contribute to stable tri-snRNP binding.

Here we dissect the role of hPrp28 in mammalian splicing *in vitro* and identify a novel intermediate of the cross-intron spliceosome assembly pathway, namely the 37S pre-B complex. Our data provide new insights into Prp28's function during splicing, and also into the initial recruitment and subsequent stable integration of the tri-snRNP during assembly of the pre-catalytic spliceosome.

## Results

### A hPrp28 mutant stalls spliceosome assembly before complex B

**Figure 1:**
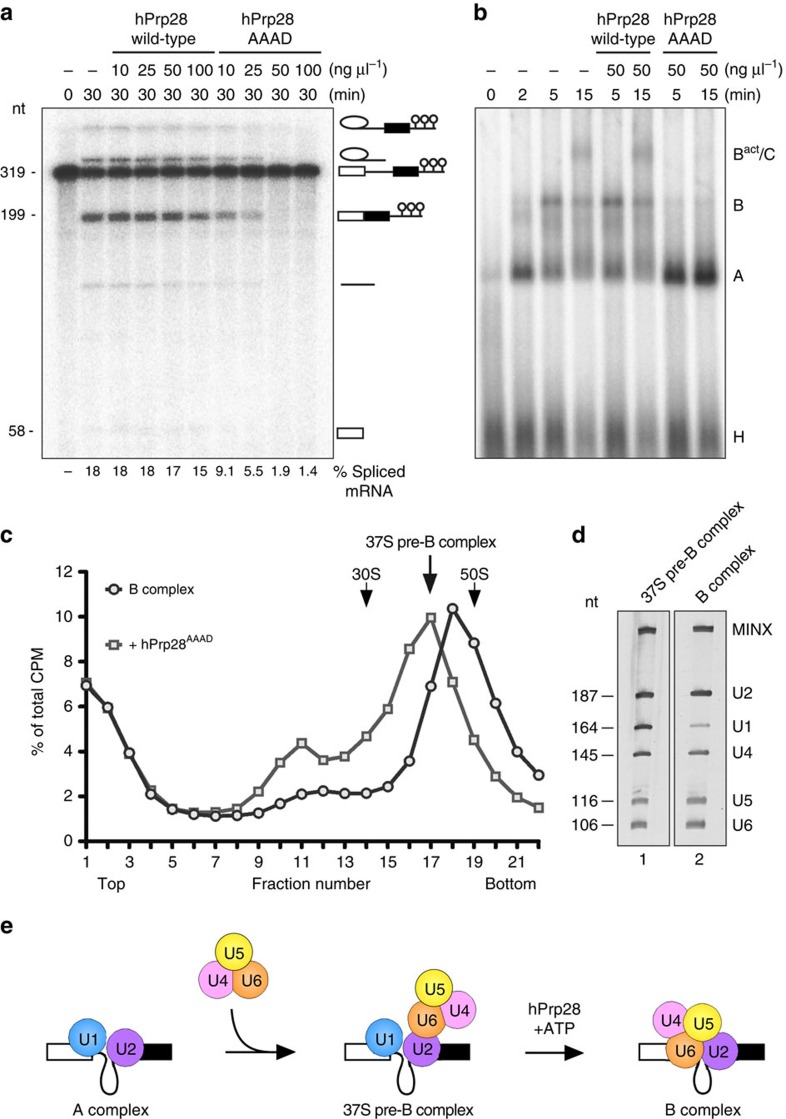
An excess of ATPase-deficient hPrp28 stalls splicing before spliceosomal B complex formation. (**a**) *In vitro* splicing of ^32^P-labelled MINX-MS2 pre-mRNA in HeLa nuclear extract in the presence of increasing amounts (10–100 ng μl^−1^) of recombinant hPrp28 or an AAAD mutant (hPrp28^AAAD^) thereof, as indicated above each lane. RNA was analysed by denaturing PAGE and visualized by autoradiography. The positions of the pre-mRNA, and splicing intermediates and products are indicated on the right. The nucleotide length of the pre-mRNA, mRNA and 5′ exon is indicated on the left. The % spliced mRNA is indicated below each lane. The average % spliced mRNA±s.d. (from three independent experiments) observed in the presence of 50 and 100 ng μl^−1^ hPrp28^AAAD^ was 2.65±0.80 and 2.05±0.77, respectively. (**b**) Analysis of splicing complexes formed in nuclear extract in the presence of 50 ng μl^−1^ hPrp28 or hPrp28^AAAD^ by agarose gel electrophoresis in the presence of heparin. The positions of H, A, B and B^act^/C complexes are indicated. (**c**) Splicing complexes were assembled on ^32^P-labelled MINX-MS2 pre-mRNA in HeLa nuclear extract for 6 min either in the absence or presence of an inhibitory concentration (50 ng μl^−1^) of the recombinant hPrp28^AAAD^ protein, and were analysed on a 10–30% glycerol gradient containing G-150 buffer. The percent of total radioactivity in each gradient fraction is plotted. Sedimentation values were determined using prokaryotic ribosomal subunits run in parallel. (**d**) Spliceosomal complexes in peak fractions (37S pre-B, fractions 16–18; B complex, fractions 17–19) were subjected to MS2 affinity selection and RNA was analysed by denaturing PAGE followed by silver staining. RNA identities are indicated on the right. Nucleotide length (nt) markers are derived from the snRNAs from purified human 37S cross-exon complexes run in parallel. (**e**) Schematic of the spliceosome assembly stages leading to a pre-catalytic B complex.

To dissect the role of the DEAD-box protein hPrp28 during human spliceosomal B complex formation, we first assayed the effect of an excess of hPrp28, in which the DEAD motif was mutated to AAAD (designated hPrp28^AAAD^), on MINX-MS2 pre-mRNA splicing *in vitro*. As amino acids from the DEAD-box motif are involved in ATP hydrolysis^32^,^33^, these mutations should abolish Prp28's ATPase activity. Because hPrp28 adopts an active conformation first when incorporated into the spliceosome^16^, the ATPase activity of recombinant hPrp28^AAAD^ could not be tested *in vitro*. However, a single alanine mutation in the DEAD motif (DEAD to AEAD) of yeast Prp28 severely reduced its ATPase activity, and yPrp28^AEAD^, as well as yPrp28^DAAD^, exhibit a lethal phenotype *in vivo*^13^. Addition of hPrp28^AAAD^, but not recombinant wild-type protein, impaired pre-mRNA splicing *in vitro* (Fig. 1a), with nearly complete inhibition of both catalytic steps observed at 50 ng μl^−1^, which corresponds to a ∼50-fold excess over the endogenous protein. Thus, hPrp28^AAAD^ acts as a dominant-negative mutant that inhibits splicing before the first catalytic step. This suggests that hPrp28^AAAD^ competes with the endogenous wild-type protein for binding but due to its inactivity, spliceosome assembly is blocked. To identify at which stage splicing is inhibited, spliceosomal complexes were assembled in the presence of a 50-fold excess of hPrp28^AAAD^ and then analysed by native gel electrophoresis in the presence of heparin. Formation of A, B and C complexes was observed in reactions lacking hPrp28^AAAD^ or containing wild-type recombinant hPrp28 (Fig. 1b). In contrast, in the presence of hPrp28^AAAD^, B complex assembly was severely reduced and A complexes accumulated (Fig. 1b). Thus, spliceosome assembly is stalled during the A to B complex transition, indicating that hPrp28 activity is required at this stage. In *S. cerevisiae* Prp28 also plays a very early role before U2 recruitment to the spliceosome^34^. However, the rate of stable U2 snRNP recruitment (that is, A complex formation) was not substantially altered in the presence of hPrp28^AAAD^ (Supplementary Fig. 1).

### Purification of a novel spliceosome assembly intermediate

To analyse the stalled spliceosomal complexes in more detail, we purified them by glycerol gradient centrifugation and subsequent MS2 affinity selection. Spliceosomes assembled in the presence of hPrp28^AAAD^ peaked in the ∼37S region of the gradient, whereas B complexes formed after 6 min of splicing without hPrp28^AAAD^ peaked at ∼45S (Fig. 1c). Affinity-purified 37S spliceosomes formed in the presence of hPrp28^AAAD^ contained stoichiometric amounts of all five snRNAs plus the MINX-MS2 pre-mRNA (Fig. 1d). In contrast, U1 snRNA was substantially underrepresented in purified B complexes (Fig. 1d). Thus, in the presence of hPrp28^AAAD^, stoichiometric amounts of U1 snRNP remain associated with the pre-mRNA, and the tri-snRNP is recruited but not yet stably bound, as evidenced by the absence of a B complex in native gels in the presence of heparin (Fig. 1b). As a complex with these characteristics has not yet been described, it represents a novel mammalian spliceosome assembly intermediate (henceforth termed the 37S pre-B complex), formed after complex A but before hPrp28 action and stable tri-snRNP integration (Fig. 1e).

**Figure 2:**
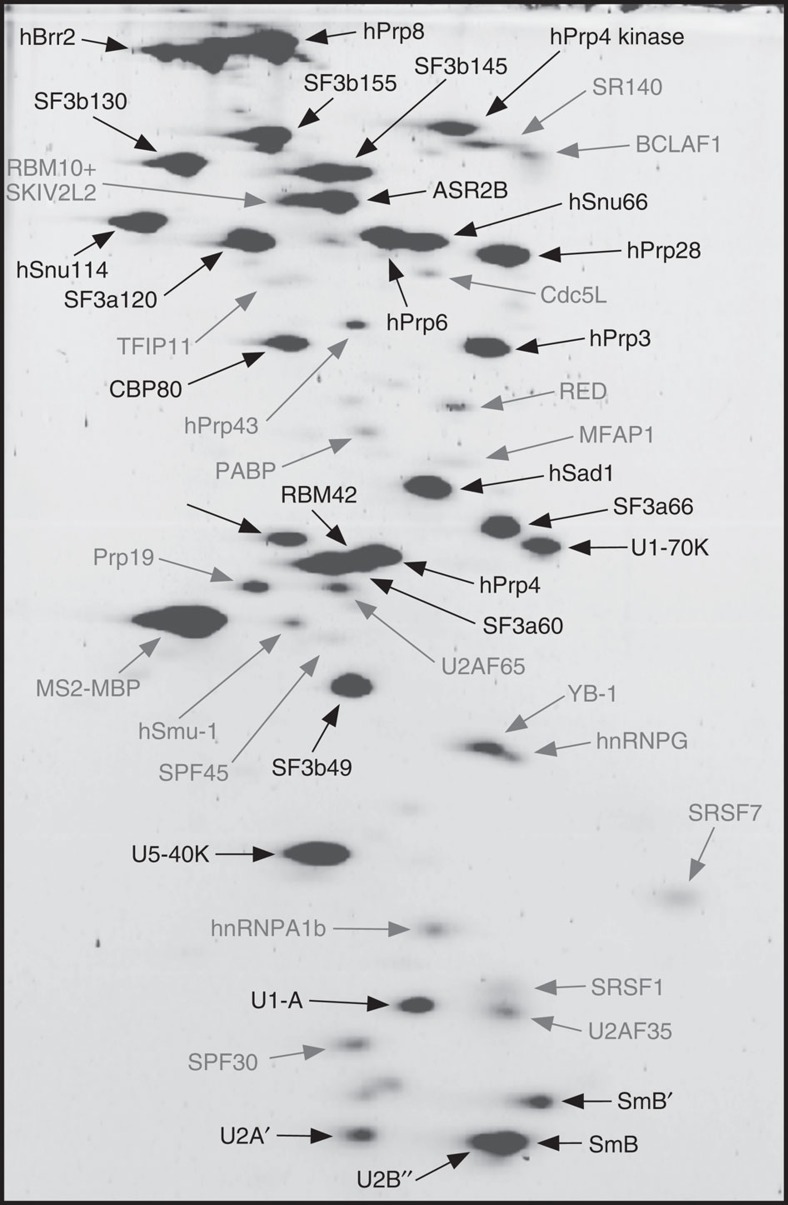
Identification of abundant proteins in purified 37S pre-B complexes. Proteins (>25 kDa) associated with affinity-purified 37S pre-B complexes were separated by 2D gel electrophoresis, stained with RuBPS, and the identities of single protein spots were determined by mass spectrometry. Abundant proteins were identified by visual inspection and are indicated in black, and less abundant ones in grey.

Mass spectrometry (MS) of affinity-purified pre-B complexes revealed that, consistent with their snRNA composition, they contain nearly all proteins associated with the U1 and U2 snRNPs, and U4/U6.U5 tri-snRNP, including hPrp28 (Supplementary Data 1). Thus, the defect in spliceosome assembly is not due to the absence of hPrp28, but presumably its lack of enzymatic activity. Indeed, western blot analyses showed that endogenous hPrp28 was quantitatively replaced by the hPrp28^AAAD^ mutant in affinity-purified pre-B complexes (Supplementary Fig. 2), indicating that hPrp28^AAAD^ outcompetes the endogenous protein for binding to the spliceosome. Compared with complex B, the pre-B complex contained lower peptide counts for the B-specific proteins RED, MFAP1, FBP21, hSmu-1, hPrp38 and hSnu23, and the Prp19/CDC5L complex and related proteins. To determine which proteins are present in stoichiometric amounts, we performed two-dimensional (2D) gel electrophoresis followed by MS. Abundant proteins (with a molecular mass above 25 kDa) in pre-B complexes included essentially all U1, U2 and U4/U6, U5 and tri-snRNP proteins plus hPrp4 kinase, CBP80 and ASR2B (Fig. 2, Table 1). The 2D analysis also confirmed that the B-specific proteins are present in very low levels. B complexes contain a similar set of abundant proteins, except that hPrp4 kinase, RBM42 and U1 snRNP proteins are no longer abundant, and most B-specific proteins, plus SRSF1, hnRNP A1 and hPrp19 are present in large amounts^26^. Thus, an excess of hPrp28^AAAD^ blocks spliceosome assembly before U1 displacement and recruitment of B-specific proteins.

hPrp6 and hPrp31 are phosphorylated by hPrp4 kinase during B complex formation^35^. As hPrp4 kinase is abundant in pre-B complexes, we assayed via western blot whether hPrp31 is phosphorylated at this stage, using antibodies specific for phosphorylated hPrp31. Only a very low level of phospho-hPrp31 was detected in purified pre-B complexes compared with B (Supplementary Fig. 2), indicating that hPrp4 kinase is activated during the pre-B to B transition.

### Analysis of the RNA–RNA network in the 37S pre-B complex

**Figure 3:**
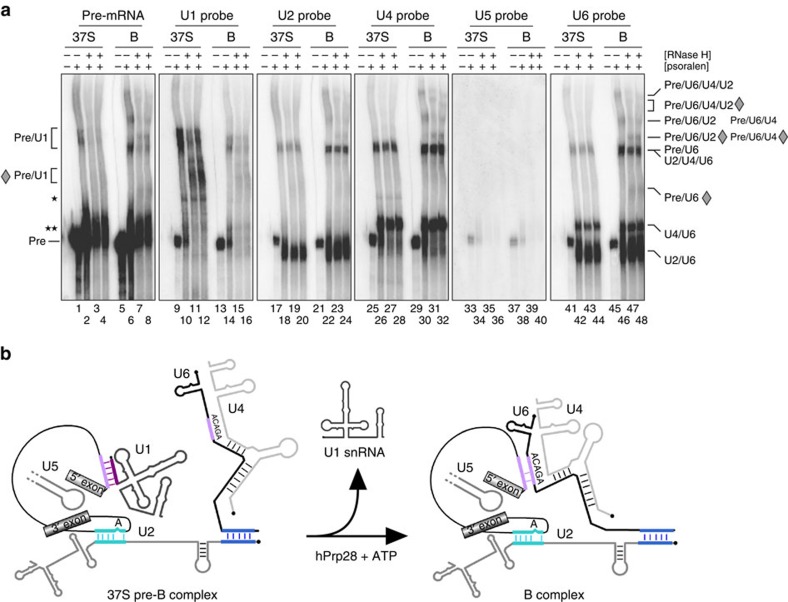
Identification of RNA–RNA interactions in the 37S pre-B complex via psoralen crosslinking. (**a**) Affinity-purified pre-B and B complexes were UV-irradiated ± psoralen (AMT) as indicated. Total psoralen-crosslinked RNA was incubated with RNase H and an oligonucleotide complementary to exon 2 of the MINX pre-mRNA as indicated above each lane. RNA–RNA crosslinks were identified by northern blot analyses, incubating sequentially with ^32^P-labelled probes against the pre-mRNA, and U1, U2, U4, U5 and U6 snRNAs. The blot was stripped of each ^32^P-probe before incubation with a subsequent probe. ^32^P-labelled MINX pre-mRNA, on which the pre-B and B complexes were formed, is visible in all panels. The lower intensity of the ^32^P-pre-mRNA in the U5 panel is due to decay of the original signal. The positions of crosslinked RNA species are indicated. Bands appearing after RNase H digestion are indicated with a diamond (◊). *A potential U1/U4 crosslink and ^**^Internally crosslinked MINX pre-mRNA. (**b**) Schematic representation of the RNA–RNA networks in the pre-B and B complexes. The 5′ss base pairing interaction with U1 followed by U6 is highlighted purple, the U2-branch site base pairing interaction is shown in light blue and U2/U6 helix II is shown in dark blue.

We next investigated RNA–RNA interactions within pre-B and B complexes by performing psoralen (4′-aminomethyl-4,5′,8-trimethylpsoralen, AMT) crosslinking. RNA–RNA crosslinks were analysed via Northern blotting by sequentially incubating with ^32^P-labelled probes against MINX pre-mRNA and the U1, U2, U4, U5 and U6 snRNAs. Several bands were detected by two or more probes, indicating crosslinking of two (or more) of these RNAs (Fig. 3a; Supplementary Fig. 3). For example, crosslinks between U2 and U6, and U4 and U6, and a triple crosslink between U2, U4 and U6 were observed in both complexes (Fig. 3a). The migration behaviour of the U2/U6 crosslink indicates formation of U2/U6 helix II (ref. 36), and thus the U4/U6.U5 tri-snRNP docks with the pre-B complex, at least in part, by interacting with U2.

Several slowly migrating bands were observed with the MINX-MS2 pre-mRNA and the snRNAs (with the exception of U5) after ultraviolet irradiation, indicating the formation of pre-mRNA/snRNA crosslinks. To confirm that these bands contained the pre-mRNA, we performed RNase H cleavage with a DNA oligonucleotide complementary to the 3′ exon of the MINX-MS2 pre-mRNA (designated exon oligo); loss or reduction in a given crosslinked species after RNase H digestion indicates the presence of pre-mRNA. Two slower migrating bands were detected with both the pre-mRNA and U1 probes in the pre-B complex but only weakly in B (Fig. 3a, lanes 1–8 and 9–16). RNAse H digestion with the exon oligo reduced their intensity and led to the appearance of two faster migrating bands that are detected by the U1 and pre-mRNA probes (Fig. 3a). This demonstrates crosslink formation between the pre-mRNA and U1, consistent with a U1/5ss interaction in the pre-B complex, and thus the requirement for hPrp28 activity for U1/5′ss unwinding. Although U2 snRNA base pairs with the BPS already in complex A^37^, a pre-mRNA/U2 crosslink was not observed with either complex, likely due to inaccessibility of the U2/BPS duplex to psoralen; indeed a U2/pre-mRNA crosslink was not observed previously with human B or B^act^ complexes^36^.

In contrast, several slowly migrating bands were observed with both the pre-mRNA and U6, U4 and U2 probes, with the B complex, but only very weakly or not at all with pre-B. These bands include pre-mRNA/U6/U4/U2, pre-mRNA/U6/U2, pre-mRNA/U6/U4 and pre-mRNA/U6 crosslinked species (Fig. 3). The latter likely co-migrates with the very strong U2/U4/U6 crosslink and is only visible after RNAse H digestion with the exon oligo (Fig. 3, lanes 46–48). Previous psoralen crosslinking studies analysing affinity-purified human B complexes formed on PM5 pre-mRNA, confirmed the formation of a U6/pre-mRNA crosslink that, due to the increased length of PM5 versus MINX pre-mRNA, no longer co-migrated with the U2/U4/U6 triple crosslink and was thus visible even without RNAse H digestion^36^. Formation of a U6/pre-mRNA crosslink (and other crosslinks containing U6, the pre-mRNA and additional snRNAs) is consistent with an interaction between the U6 ACAGA box and intron nucleotides near the 5′ss. Thus, in the 37S pre-B complex the U1 snRNP is still base paired at the 5′ss, while the U4/U6.U5 tri-snRNP associates, at least partially, via U2/U6 helix II (Fig. 3b). In the B complex, U1 snRNA is displaced from the 5′ss and U6 snRNA interacts with intron nucleotides near the 5′ss.

### Pre-B complexes can be chased into active spliceosomes

**Figure 4:**
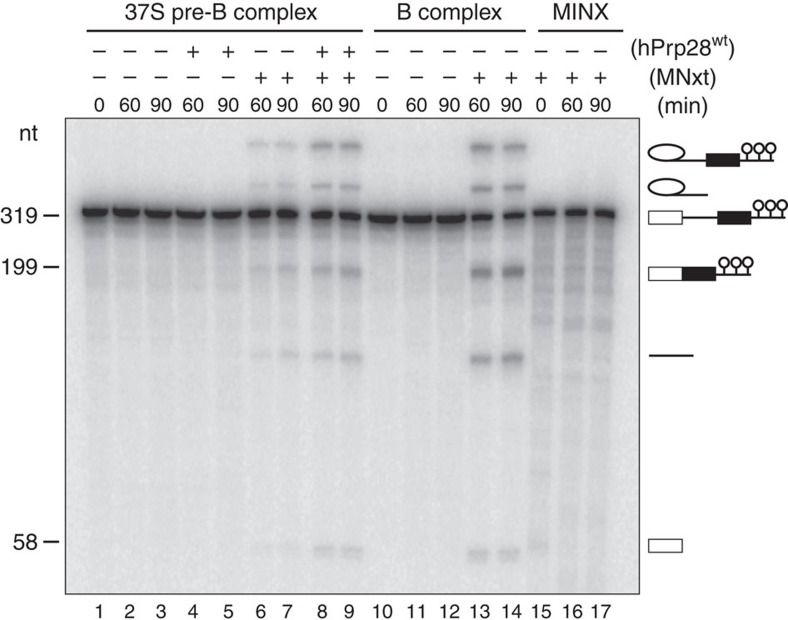
Purified 37S pre-B complexes can be chased into catalytically active spliceosomes. Affinity-purified pre-B or B complexes, or MINX-MS2 pre-mRNA (as indicated above) were incubated at 30 °C for the indicated times (0–90 min) under splicing conditions in the presence of buffer alone (lanes 1–3 and 10–12) or micrococcal nuclease-treated HeLa nuclear extract (MNxt; lanes 6–9 and 13–17). To outcompete the hPrp28^AAAD^ mutant present in the pre-B complexes, 50 ng μl^−1^ of recombinant hPrp28^wt^ protein was added before incubation at 30 °C (lanes 4–5 and 8–9). RNA was analysed by denaturing PAGE and visualized with a Phosphorimager. The positions of the pre-mRNA, splicing intermediates and products are indicated on the right. Nucleotide length (nt) markers are derived from the snRNAs from purified human 37S cross-exon complexes run in parallel.

To determine whether the hPrp28^AAAD^-stalled pre-B complex is a functional assembly intermediate, we performed chase experiments. When affinity-purified pre-B complexes were incubated under splicing conditions in the absence of extract, or after addition of hPrp28^wt^ alone, no splicing was observed (Fig. 4, lanes 1–5). However, when micrococcal nuclease (MN)-treated nuclear extract—in which all endogenous snRNPs were degraded—was added, splicing was observed, albeit at a lower level than observed with purified B complexes plus MN-extract (Fig. 4, lanes 6–7 versus 13–14; where the average splicing product formation (from two independent experiments)±the s.d. was 3.9±0.5% for pre-B versus 18.4±2.6% for B after 60 min). Thus, purified pre-B complexes can be chased into mature spliceosomes that catalyse both steps of splicing. Splicing was not observed when pre-mRNA alone was incubated under splicing conditions with MN-treated extract (Fig. 4, lanes 15–17). The efficiency of the pre-B complex chase, but not that of B complexes, could be improved by adding an excess recombinant hPrp28^wt^ protein to the reaction (Fig. 4, lanes 8–9 and Supplementary Fig. 4; 9.6±2.9% splicing product formation with pre-B plus MN extract and hPrp28 after 60 min). This suggests that the lower splicing efficiency of pre-B complexes plus MN extract results from the inability of endogenous hPrp28 in the MN-treated extract to efficiently displace hPrp28^AAAD^.

### The structures of the pre-B and B complex are different

**Figure 5:**
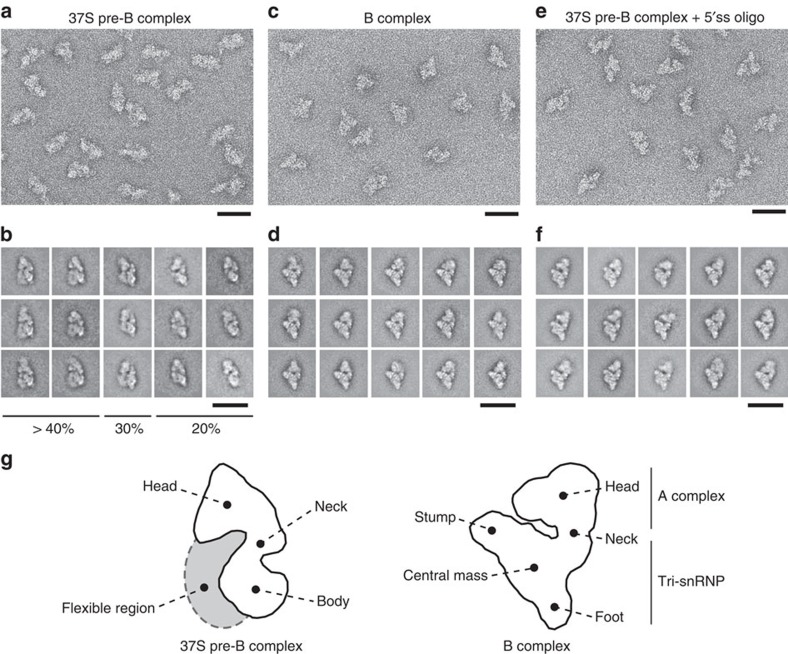
Electron microscopy of the 37S pre-B complex. Overviews of negatively stained, affinity-purified 37S pre-B (**a**) and B complexes (**c**) or affinity-purified 37S pre-B complexes incubated solely with the 5′ss RNA oligo (**e**). Representative class averages from 9,000 to 11,000 single-particle images of each complex are shown in the galleries in **b**,**d** and **f**. Scale bars, 50 nm. (**g**) Schematic representation of the 37S pre-B complex and B complex, with main structural features labelled according to Boehringer *et al*.^52^

The 37S pre-B and 45S B complexes exhibit very different sedimentation coefficients despite having very similar molecular masses, suggesting that they differ structurally. We thus analysed affinity-purified pre-B complexes stalled in the presence of hPrp28^AAAD^ by negative-stain electron microscopy (EM) after gradient fixation (GraFix; Fig. 5). Well defined, single particles were visible in the EM overview (Fig. 5a). Classification and class averaging of single particle images (Fig. 5b) revealed that the pre-B complex possesses an upper head domain and an elongated body domain, and has a maximum length of ∼40 nm. The head domain is triangular and appears similar in all classes. In contrast, the lower body domain shows significant differences in its structure, varying in size and form in almost all class averages, suggesting it is flexible or structurally heterogeneous (Fig. 5b). However, the different structures could potentially also reflect different orientations of the complex on the EM grid. In contrast, B complexes appear structurally homogenous with a characteristic orientation in almost all class averages and clearer structural details (Fig. 5c,d). Overall the B complex appears more compact with less conformational flexibility of the head domain compared with the pre-B complex, which might contribute to its higher S-value. The structure of pre-B in most classes is very different from that of B, indicating that the pre-B to B complex transition is accompanied by a substantial structural rearrangement.

### Tri-snRNP stabilization via addition of a 5′ss RNA *in trans*

**Figure 6:**
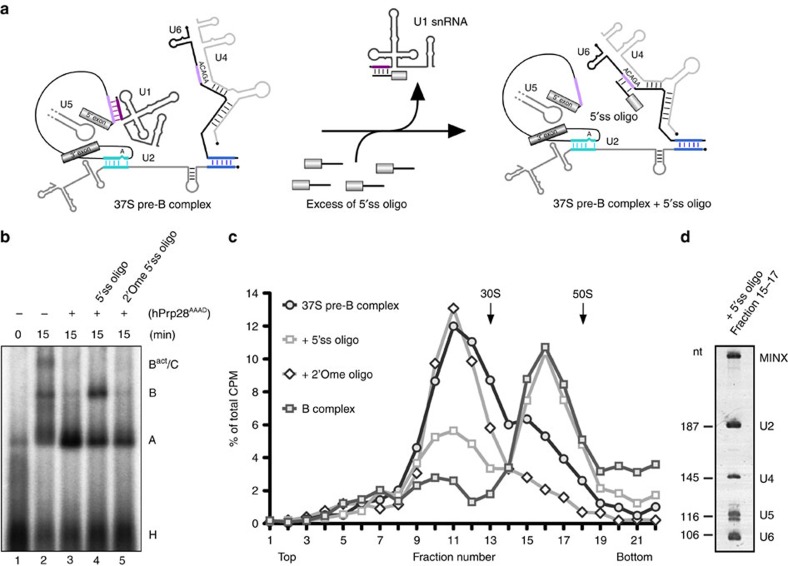
Addition of a 5′ss RNA oligonucleotide to 37S pre-B complexes induces stable tri-snRNP binding. (**a**) Schematic of the effect of the addition of an excess of a 5′ss containing oligonucleotide on RNA–RNA interaction within the pre-B complex. Colouring as in Fig. 3. (**b**) 37S pre-B complexes were assembled in nuclear extract on ^32^P-labelled MINX-MS2 pre-mRNA in the presence of an inhibitory concentration of hPrp28^AAAD^. After 3 min, wild type or 2′Ome 5′ss oligo was added at an 100-fold excess. Splicing complex formation was analysed on an agarose gel in the presence of heparin and visualized by autoradiography. The positions of H, A, B and B^act^/C complexes are indicated. (**c**) Glycerol gradient centrifugation (150 mM KCl) of affinity-purified 37S pre-B complexes alone or after incubating with either a wild-type 5′ss RNA oligonucleotide or a 2′Ome version thereof. Affinity-purified B complexes were run in parallel. The percent of total radioactivity (^32^P-MINX pre-mRNA) is plotted for each gradient fraction. (**d**) RNA was recovered from the indicated peak fractions, separated by denaturing PAGE and visualized by silver staining. Identities of snRNAs are indicated. Nucleotide length (nt) markers are derived from the spliceosomal snRNAs from purified human 37S cross-exon complexes run in parallel.

We next tried to bypass the exchange of U1 for U6 at the 5′ss, which is blocked in the pre-B complex due to the presence of hPrp28^AAAD^, by adding an excess of an RNA oligonucleotide containing an optimized 5′ss sequence (designated 5′ss oligo). This 5′ss oligo is complementary to the 5′ end of U1 snRNA, and to U6 snRNA (Supplementary Fig. 5a). When added in excess, it not only competes with the 5′ss for U1 binding, thereby disrupting the U1/5′ss interaction, but also interacts with the U4/U6.U5 tri-snRNP, in particular U6 snRNA^27^,^38^ (Fig. 6a). However, a 2′O-ribose methylated (2′Ome) version of this 5′ss oligo does not bind the tri-snRNP, and only leads to U1 displacement from the endogenous 5′ss (ref. 38). When we assembled spliceosomes in nuclear extract in the presence of an inhibitory concentration of hPrp28^AAAD^ and subsequently added an excess of the 2′Ome 5′ss oligo, no B complex formation was observed (Fig. 6b). However, addition of the non-methylated form of the 5′ss oligo led to B complex formation (Fig. 6b). To determine whether U1 was underrepresented in the spliceosomal complexes formed after addition of the wild-type and 2′Ome 5′ss oligo, we affinity-purified them. Analysis of their RNA compositions showed a clear reduction in U1, but not other snRNAs, comparable to that observed for U1 with the B complex (Supplementary Fig. 5b). Using ^32^P end-labelled 5′ss RNA oligos, we also confirmed that the wild-type oligo, but not the 2′Ome version, binds to affinity-purified pre-B complexes (Supplementary Fig. 6), consistent with the former interacting with components of the tri-snRNP. Thus, displacement of U1 from the pre-mRNA's 5′ss in the absence of hPrp28 activity is not sufficient to allow the loosely bound tri-snRNP in the pre-B complex to stably interact with the 5′ss of the pre-mRNA.

We next tested whether stable tri-snRNP association can be induced by the 5′ss oligo in the absence of nuclear extract. When affinity-purified, pre-B complexes were analysed on a glycerol gradient containing 150 mM KCl (Fig. 6c), the majority of the complexes peaked in fractions 10–12 with an *S*-value<30S (Fig. 6c), consistent with dissociation of the U4/U6.U5 tri-snRNP under these conditions. Thus, affinity-purified pre-B complexes (that is, in the absence of nuclear extract) are not stable when subjected to a second glycerol gradient containing 150 mM salt. However, addition of the 5′ss oligo, but not the 2′Ome form, led to the formation of a complex with a higher *S*-value that co-migrated with purified B complexes run in parallel. Affinity selection of this stabilized complex showed that it contains stoichiometric amounts of the U2 snRNP and U4/U6.U5 tri-snRNP, and the MINX-MS2 pre-mRNA, based on its RNA composition (Fig. 6d). Thus, addition of a 5′ss oligo *in trans* leads to a more stably associated tri-snRNP and a shift in *S*-value even in the absence of extract, indicating that all factors required for tri-snRNP stabilization are already present in purified pre-B complexes. Thus, the B-specific proteins, which are for the most part absent from the pre-B complex, are not required for salt-stable tri-snRNP integration.

EM of affinity-purified pre-B complexes incubated with the 5′ss oligo in the absence of extract, showed well-defined single particles (Fig. 5e). The most frequently observed classes are very similar and show only a low degree of structural heterogeneity (Fig. 5f). The 5′ss oligo stabilized pre-B complex adopts a structure almost identical to that of the B complex (Fig. 5c,d), with nearly no structural similarities with the pre-B complex (Fig. 5a,b). Thus, addition of an excess of a 5′ss *in trans* induces a significant change in the structure of the 37S pre-B complex such that it adopts an organization characteristic for B complexes with stably integrated tri-snRNP. This is consistent with the idea that the interaction of the tri-snRNP with a 5′ss *in trans* mimicks the situation with the pre-mRNA, where the 5′ss is present *in cis.* Taken together, our data indicate that a decisive factor for stable binding of the tri-snRNP during B complex formation is its interaction with the 5′ss.

## Discussion

Here we identify a spliceosome assembly intermediate formed before hPrp28 action, which to date has not been characterized in higher eukaryotes. Although the 37S pre-B complex contains stoichiometric amounts of all five snRNAs, the tri-snRNP is not stably associated, indicating that association of the latter can occur before hPrp28 action, but its stable integration into the spliceosome requires ATP hydrolysis by hPrp28 (Fig. 1). The pre-B complex most likely previously escaped detection because it is relatively unstable and/or in the presence of wild-type hPrp28 is rapidly converted to a heparin-stable, spliceosomal B complex. Importantly, chase experiments with affinity-purified pre-B complexes and MN-treated nuclear extract (Fig. 4), demonstrated that it is a functional spliceosome assembly intermediate.

Affinity-purified pre-B complexes contained essentially all U1, U2, U4/U6 and U5 snRNP proteins (Fig. 2; Table 1; Supplementary Data 1). However, unlike the B complex, the pre-B complex contains stoichiometric amounts of hPrp4 kinase, RBM42 and U1 snRNP proteins, but only very low levels of the B-specific proteins RED, MFAP1, FBP21, hSmu-1, hPrp38 and hSnu23. As hPrp4 kinase is not present in human spliceosomal A complexes and very little is detected in the B complex^26^, this kinase appears to associate transiently with the spliceosome. Human Prp4 kinase phosphorylates the tri-snRNP proteins hPrp31 and hPrp6 during B complex formation, and is required for stable B complex formation *in vitro*^35^. As hPrp31 was only weakly phosphorylated in affinity-purified pre-B complexes (Supplementary Fig. 2), hPrp4 kinase appears to be inactive at this stage of spliceosome assembly. What triggers hPrp4 kinase activity and ultimately leads to its dissociation from the B complex is not clear. One possibility is that the conformational rearrangement that occurs during the pre-B to B transition (see below) might trigger hPrp4 kinase activity (or vice versa) and at the same time induce structural changes that result in its release. Consistent with this, addition of the 5′ss RNA oligo to extracts where pre-B complexes were first allowed to form (in the presence of hPrp28^AAAD^), leading to stable tri-snRNP intergration and formation of a 45S complex, also leads to enhanced phosphorylation of hPrp31 (Supplementary Fig. 2).

The structure of the 37S pre-B complex differs from that of the 45S B complex (Fig. 5), indicating that a substantial rearrangement occurs before or during stable tri-snRNP integration during intron-defined spliceosome assembly. A structural change was also recently observed when a cross-exon splicing complex with loosely associated tri-snRNP was converted into a B-like complex with stably associated tri-snRNP^38^. Previously, immuno-EM studies indicated that the U2 snRNP is in the head domain of the B complex^39^, while U5 snRNP and U4/U6 snRNP are in the body and neck region, respectively^40^. Unfortunately, at the current resolution, it is not possible to assign the probable positions of the snRNPs to the 2D images of the pre-B complex. Initial attempts to localize the U2 or tri-snRNP via immuno-EM were unsuccessful due to the labile nature of the pre-B complex. Thus, it is presently not clear whether the tri-snRNP itself undergoes an RNP rearrangement or alternatively that its position relative to components of the A complex has changed.

**Figure 7:**
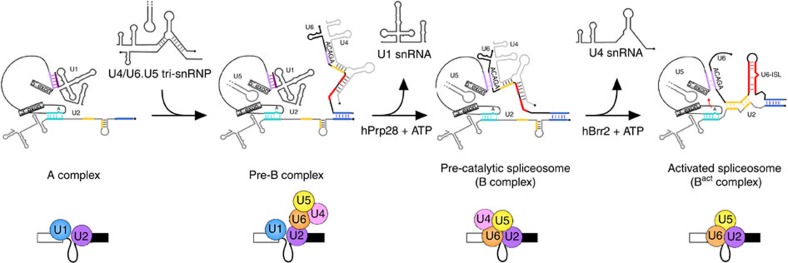
Summary of the cross-intron spliceosome assembly pathway. The A, pre-B, B and B^act^ complexes and their RNA–RNA networks are shown schematically. The 5′ss base pairing interaction with U1 followed by U6 is highlighted purple, the U2-branch site base pairing interaction is shown in light blue, U2/U6 helix II is shown in dark blue and U2/U6 helix I is shown in yellow.

The identification and characterization of an assembly intermediate formed after A but before the pre-catalytic B complex, allows a more clear definition of what precisely a spliceosomal B complex is. Namely, our data demonstrate that the formation of a B complex requires ATP binding/hydrolysis by Prp28, during which U1 is displaced from the 5′ss and replaced by U6 snRNA. At the same time a structural rearrangement occurs which leads to stable association of the tri-snRNP and recruitment of the B-specific proteins. The U4 and U6 snRNAs are still base-paired in the pre-catalytic B complex, keeping U6 in an inactive state^41^. The displacement of U1 by Prp28 occurs before or during B complex formation, whereas release of U4 by the helicase Brr2 occurs during the subsequent spliceosome activation step (Fig. 7). Thus, these two spliceosomal helicases act sequentially and, although displacement of U1 from the 5′ss may be a prerequisite for activation, they apparently do not necessarily act in a coordinated fashion.

Interestingly, the 37S pre-B complex, which is an early intermediate of the cross-intron spliceosomal assembly pathway, shares similarities but also differences with the recently described 37S cross-exon complex^27^,^38^. The latter forms on an exon containing solely flanking intron sequences, and is an early intermediate of the cross-exon spliceosome assembly pathway. Both complexes share nearly identical protein and snRNA compositions, containing U1 and U2 snRNP, and loosely associated tri-snRNP (Fig. 2)^38^. However, despite nearly identical compositions, each complex appears to have a distinct morphology as evidenced by EM (Supplementary Fig. 7). This apparent structural difference might reflect the different location of U1 snRNP relative to the BPS-bound U2 snRNP in both complexes; U1 is base-paired with the 5′ss upstream of the BPS in the 37S pre-B complex, whereas in the 37S cross-exon complex it is bound to the 5′ss downstream of the BPS and the exon. It will be interesting to compare the three-dimensional structure of these complexes by cryo-EM in the future, to more clearly elucidate any structural differences.

Stable tri-snRNP association with the 37S cross-exon complex can be achieved by adding a 5′ss-containing RNA oligonucleotide *in trans*—either to splicing extract or to affinity-purified cross-exon complexes, and is accompanied by a major structural change that generates a complex (the so-called B-like complex) with a morphology highly similar to the B complex (Supplementary Fig. 5)^27^,^38^. We observed a similar effect with 37S pre-B complexes. Namely, addition of the 5′ss oligo to splicing extracts containing inhibitory concentrations of hPrp28^AAAD^ or to affinity-purified 37S pre-B complexes led to the formation of a complex with stably associated tri-snRNP (Fig. 6), which appears to be structurally very similar to the B complex, as evidenced by EM (Fig. 5; Supplementary Fig. 7). This is consistent with the idea that the 37S complexes with loosely associated U4/U6.U5 tri-snRNP represent a very similar intermediate in both the exon-defined and intron-defined spliceosome assembly pathways, and that stable tri-snRNP integration is triggered by its interaction with the pre-mRNA's 5′ss in both pathways. They further suggest that the exon- and intron-defined spliceosome assembly pathways converge at the B complex stage. Indeed, as splicing catalysis can only occur across an intron, cross-exon complexes must be converted to cross-intron complexes before catalysis.

As affinity-purified pre-B complexes contain only very low levels of B-specific proteins, we conclude that they are not essential for stable integration of the U4/U6.U5 tri-snRNP during B complex formation. Transformation of 37S cross-exon complexes with loosely associated tri-snRNP, to B-like complexes with stably integrated tri-snRNP, also did not require B-specific proteins, indicating that they also do not contribute substantially to stable tri-snRNP binding during this transition^38^. Instead, B-specific proteins appear to function after B complex formation and thus likely contribute to the subsequent activation step, during which they are displaced from the spliceosome.

Our data provide important insights into hPrp28′s role during mammalian splicing. Human Prp28 is still present in stoichiometric amounts in affinity-purified pre-B complexes and the vast majority that is present is the hPrp28^AAAD^ mutant (Fig. 2; Supplementary Fig. 2). Thus, the latter outcompetes the endogenous wild-type protein for binding to the spliceosome. Given that the DEAD to AAAD exchange has little or no effect on the overall structure of the mutant protein, it is likely that it is incorporated in the spliceosome in an identical manner, establishing the same RNA and protein contacts. Thus, the defect in spliceosome assembly is not due to the absence of hPrp28 nor apparently to its aberrant incorporation into the tri-snRNP/spliceosome, but rather most likely due to its lack of ATPase/unwinding activity.

The hPrp28^AAAD^-mediated block in spliceosome assembly occurs before disruption of the U1/5′ss interaction (Fig. 3). Thus, ATP hydrolysis by hPrp28 is also a prerequisite for displacement of U1 from the 5′ss in higher eukaryotes. As the U1/5′ss and U6/5′ss interactions are mutually exclusive, the latter interaction can only occur within the spliceosome if U1 has vacated the 5′ss. Interestingly, addition of a 2′Ome 5′ss oligo to a splicing reaction containing inhibitory amounts of hPrp28^AAAD^, did not support B complex formation (Fig. 6), even though it led to displacement of the majority of U1 from the pre-mRNA (Supplementary Fig. 5). Thus, an accessible 5′ss present *in cis* is apparently not sufficient to trigger stable tri-snRNP integration nor the structural change converting the spliceosome from a 37S to 45S complex. This suggests that in the spliceosome hPrp28 may play an additional role. Crosslinking studies showed that in the human B complex, the ATP-binding site of hPrp28 is close to the 5′ss, just a few nucleotides downstream of the exon–intron junction, which is contacted by hPrp8, and that ATP hydrolysis is required for hPrp28 interaction with the 5′ss (ref. 31). It was thus proposed that hPrp28 not only unwinds the U1/5′ss duplex but also facilitates a conformational change that positions the 5′ss for base pairing with the U6 ACAGA box^31^. Although this coordinated handoff appears to be circumvented, at least partially, by adding an excess of an unmodified 5′ss containing oligo that can base pair with U6 in the absence of Prp28 activity and thereby trigger stable tri-snRNP binding (Fig. 6), this is not likely the case within the spliceosome. Recent studies investigating stable tri-snRNP integration after its initial association with a cross-exon spliceosomal complex, indicated that a major determinant for stable tri-snRNP integration is the interaction of Prp8 with the 5′ss (ref. 38). Thus, hPrp28 action may also position the 5′ss for interaction with Prp8, leading to stable tri-snRNP integration and formation of the B complex. Consistent with this, *S. cerevisiae* Prp28 interacts genetically with Prp8 (ref. 42). Such a coordinated handover of the 5′ss from U1 to hPrp8/U6 by hPrp28 potentially contributes to the high fidelity of 5′ss recognition. Indeed, genetic studies in yeast indicate that Prp28 proofreads the 5′ss and ultimately rejects poor 5′ splice sites^15^.

Human Prp28 adopts an active conformation first when incorporated into the spliceosome^16^. Our data suggest that initial docking of the tri-snRNP to the A complex may already trigger a conformational change in Prp28, thereby activating it, as hPrp28′s ATPase activity is required for the subsequent assembly step that generates complex B. As the transition from a pre-B to B complex involves a major structural change (Fig. 5), which appears to require the interaction of Prp8/U6 with the 5′ss (ref. 38; Fig. 6), it is tempting to speculate that this structural change is mediated/triggered by hPrp28′s ATPase/unwinding activity.

## Methods

### Purification of recombinant hPrp28 protein

N-terminally His_6_-tagged hPrp28 and hPrp28^AAAD^ were expressed in *Escherichia coli* Rosetta 2 (DE3) cells^20^. Cells were grown to an OD_600_ of 0.4–0.8 at 30 °C and expression of the recombinant protein was induced by adding 0.3 mM IPTG. After incubation at 18 °C overnight, cells were disrupted in 50 mM Tris-HCl pH 7.5, 2 M LiCl, 5% (v/v) glycerol and 2 mM 2-mercaptoethanol in the presence of a protease inhibitor cocktail (ALP) using a microfluidizer (M-110S, *Microfluidics*). After centrifuging at 35,000*g* for 30 min, the supernatant was loaded onto a Ni-NTA Sepharose column (Protino, Macherey and Nagel) in buffer containing 50 mM Tris/HCl pH 7.5, 500 mM NaCl, 5% (v/v) glycerol, 2 mM 2-mercaptoethanol and 10 mM imidazole. After washing, His_6_-hPrp28 or His_6_-hPrp28^AAAD^ was eluted by gradually increasing the concentration of imidazole to 300 mM. After sample concentration, purification to homogeneity was achieved by size exclusion chromatography (Superdex 200, GE Healthcare) performed in 10 mM Tris-HCl pH 7.5, 500 mM NaCl, 5% (v/v) glycerol and 2 mM 2-mercaptoethanol.

### Pre-mRNA splicing and splicing complex formation

Splicing reactions contained 40% (v/v) HeLa nuclear extract^43^, 65 mM KCl, 3 mM MgCl_2_, 2 mM ATP, 20 mM creatine phosphate and 10 nM uniformly ^32^P-labelled, m^7^G-capped MINX-MS2 pre-mRNA, and were incubated at 30 °C for the indicated times. To test the effect of recombinant hPrp28 on splicing, reactions were supplemented with His_6_-tagged wild-type hPrp28 or mutant hPrp28^AAAD^ to a final concentration of 10–100 ng μl^−1^ before the addition of the pre-mRNA and preincubated for 30 min at 30 °C to allow an exchange of the recombinant protein with the endogenous hPrp28. Splicing was initiated by addition of the radiolabelled MINX-MS2 pre-mRNA. RNA was recovered at the indicated time points and separated on a 14% polyacrylamide gel containing 6 M urea. Unspliced pre-mRNA, and splicing intermediates and products were detected using a Typhoon phosphoimager (GE Healthcare). The pre-mRNA, and splicing intermediates and products (spliced mRNA and excised lariat intron) were quantified using ImageQuantTL (GE Healthcare). The percent spliced mRNA was calculated by dividing the amount of mRNA by the amount of pre-mRNA, lariat-intermediate and splicing products (minus background), and multiplying by 100. To investigate the effect of a 5′ss-containing RNA added *in trans*, splicing reactions containing an inhibitory concentration (50 ng μl^−1^) of hPrp28^AAAD^ were first incubated for 3 min at 30 °C. The reaction was then supplemented with an RNA oligonucleotide containing a 5′ss (5′- AAG/GUAAGUAU -3′, Eurofins MWG Operon) or a 2′O-ribose methylated version thereof, to a final concentration of 1 μM followed by an additional incubation at 30 °C for 15 min. Spliceosomal complexes were analysed on a 2% (w/v) low melting point agarose gel in the presence of 0.65 mg ml^−1^ heparin^44^ and bands were visualized with a Typhoon phosphoimager (GE Healthcare). Alternatively, splicing reactions were loaded onto a linear 10–30% (v/v) glycerol gradient containing G-150 buffer (20 mM Hepes-KOH pH 7.9, 1.5 mM MgCl_2_, 150 mM KCl) or G-75 buffer (G-buffer with 75 mM KCl) as indicated, and centrifuged at 488,576*g* (*r*_max_) for 135 min at 4 °C in a Sorvall TH660 rotor. The gradients were harvested manually in 175 μl fractions from the top and the distribution of ^32^P-labelled MINX pre-mRNA was determined by Cherenkov counting.

### Affinity purification of spliceosomal complexes

*In vitro* assembled spliceosomal complexes were purified by gradient centrifugation, followed by MS2 affinity selection^25^,^38^. For this purpose, the ^32^P-labelled, MINX-MS2 pre-mRNA was incubated with a 20-fold molar excess of purified MS2-MBP fusion protein for 30 min at 4 °C before addition to the splicing reaction. B complexes were isolated after incubating a standard splicing reaction for 6 min at 30 °C; thus these kinetically stalled B complexes also contain low amounts of complexes formed before and after B, including pre-B and B^act^ complexes, that migrate in the same position or close to B complexes on the glycerol gradient. Pre-B complexes were isolated after preincubation with 50 ng μl^−1^ of hPrp28^AAAD^ protein. The splicing reactions were loaded onto a linear 10–30% (v/v) glycerol gradient containing G-150 buffer and centrifugation was performed at 112,000*g* (*r*_max_) for 15 h at 4 °C in a Sorvall TST 41.14 rotor. Gradients were harvested manually in 500 μl fractions from top to bottom. The distribution of ^32^P-labelled MINX-MS2 pre-mRNA across the gradient was determined by Cherenkov counting. Peak fractions containing spliceosomal complexes were pooled and loaded onto a column containing 150 μl (packed volume) of amylose beads (NEB). After washing with G75 buffer, bound complexes were eluted with 300 μl of G-75 buffer containing 20 mM maltose. RNA was recovered from the purified complexes, separated on a denaturing polyacrylamide gel and visualized by silver staining.

### 2D gel electrophoresis and mass spectrometry

Affinity-purified, 37S pre-B complexes were concentrated by sedimentation at 700,000*g* and RNA was subsequently digested with RNase T1, RNase A and RNase I in the presence of urea^26^. After treatment with iodoacetamide to prevent protein oxidation, the sample was concentrated by dialysis against Slide-A-Lyser Concentration solution (Pierce) and loaded onto the first dimension. The first dimension gel contained 2% (w/v) acrylamide, 1.4% methylene bisacrylamide, 7 M urea, 4 M thiourea, 40% (v/v) formamide in 40 mM Bis-TRIS-OAc pH 5.7 (ref. 26). The second dimension gel contained 8% (v/v) acrylamide. Proteins were stained with RuBPS (RubiLAB) or, for mass spectrometry, with Coomassie. Protein spots were cut out of the 2D gel and proteins were digested in-gel with trypsin. Peptides were extracted and then analysed in a liquid-chromatography-coupled electrospray ionization quadruple time-of-flight mass spectrometer (LTQ Oribitrap XL) under standard conditions. Proteins were identified by searching fragment spectra against the NCBI nonredundant database using Mascot as a search engine.

### Psoralen crosslinking

Affinity-purified spliceosomal complexes (formed on ^32^P-labelled MINX pre-mRNA) in G150 buffer were supplemented with 40 μg ml^−1^ of AMT hydrochloride^45^. After incubating for 10 min on ice, samples (±AMT) were irradiated with 365 nm ultraviolet light for 30 min at 4 °C with a distance of 4 cm between the samples and ultraviolet lamp. To further characterize the crosslinked RNA species, a 300- or 600-fold molar excess of a DNA oligo (5′- GTCCTCAACCGCGAG -3′, Eurofins MWG Operon), complementary to nucleotides 1–15 of the second exon of the MINX-MS2 pre-mRNA, and 0.1 U μl^−1^ RNase H (NEB), were added to the crosslinked RNAs and the reaction was incubated for 1 h at 37 °C. RNA was resolved on a 5% polyacrylamide gel containing 6 M urea and transferred to a nylon membrane (Hybond XL, GE Healthcare). The membrane was sequentially hybridized with ^32^P-labelled probes against the pre-mRNA, and the U1, U2, U4, U5 and U6 snRNAs. Before incubation with a different probe, ^32^P-labelled probe was removed by boiling the membrane for 30 min in 15 mM NaCl, 1.5 mM sodium citrate and 0.1% (w/v) SDS; efficient probe removal was controlled using a Typhoon phosphoimager (GE Healthcare). The snRNA composition of the affinity-purified pre-B and B complexes used for psoralen crosslinking was checked via silver staining to ensure the identity and quality of the analysed complexes.

### Chasing of affinity-purified pre-B complexes

HeLa nuclear extract was incubated with 0.5 units per μl of micrococcal nuclease (USB Affimetrix) and 1.6 mM CaCl_2_ at 30 °C for 6 min. To inactivate the MN, EGTA was added to a final concentration of 4.5 mM. Affinity-purified 37S pre-B or 45S B complexes formed on ^32^P-labelled MINX-MS2 pre-mRNA were incubated with splicing buffer alone (70 mM KCl, 3 mM MgCl_2_, 2 mM ATP, 20 mM creatine phosphate), 50 ng μl^−1^ recombinant hPrp28^wt^ or additionally in the presence of 30% (v/v) MN-treated HeLa nuclear extract ±50 ng μl^−1^ recombinant hPrp28^wt^ (as indicated). A 10-fold excess of unlabelled MINX-MS2 pre-mRNA was added to exclude the reassembly of snRNPs on the radiolabelled pre-mRNA in the event that they dissociate from the affinity-purified 37S pre-B complexes. After incubation for 30 min on ice, the reaction was incubated at 30 °C for 0–90 min. RNA was recovered, separated on a 14% polyacrylamide gel containing 6 M urea, and visualized with a Typhoon phosphoimager (GE Healthcare).

### Stabilization of affinity-purified 37S pre-B complexes

MS2 affinity-purified 37S pre-B complexes in G-75 buffer were first supplemented with a 100-fold excess of either the 5′ss RNA oligonucleotide or its 2′O-ribose methylated version, and then incubated for 15 min on ice. The reaction was loaded onto a linear 10–30% (v/v) glycerol gradient containing G-150 buffer, and centrifuged at 488,576*g* (*r*_max_) for 135 min at 4 °C in a Sorvall TH660 rotor. The gradients were harvested manually in 175 μl fractions from the top and peak fractions were subjected to a second MS2 affinity selection. RNA was recovered from the affinity-purified complexes, separated on a denaturing polyacrylamide gel and visualized by silver staining.

### Electron microscopy

For electron microscopy, affinity-purified complexes were subjected to a second, linear 10–30% (v/v) glycerol gradient containing G-75 (37S pre-B complex) or G-150 (B complex and purified 37S pre-B+5′ss oligo complex) buffer and 0-0.1% (v/v) glutaraldehyde^46^. The samples were centrifuged at 488,576*g* (*r*_max_) for 2 h in a Sorvall TH660 rotor at 4 °C and gradients were harvested manually in 175 μl fractions from the top. Particles were negatively stained by the single-carbon film method^47^. Images were recorded at a magnification of × 88,000 at 160 kV with a CM200 FEG electron microscope (Philips, The Netherlands) at room temperature and two-fold binning on a 4k × 4k CCD camera (TVIPS, Germany). For each data set, 9,000–11,000 individual single-particle images were collected and single-particle image-processing was performed using the software package IMAGIC-5 (ref. 48). Image-processing consisted of a reference-free alignment, after which the images were subjected to multivariate statistical analysis and classification^49^,^50^,^51^. The resulting class averages were used as reference images for subsequent rounds of alignment until the class averages were stable.

### Western blotting

For western blotting, proteins of affinity-purified complexes were separated by SDS–polyacrylamide gel electrophoresis (PAGE) and transferred to a nitrocellulose membrane (Protran, Whatman). The membrane was incubated in TBS buffer (50 mM Tris-HCl pH 7.5, 150 mM NaCl, 1% (v/v) Tween-20) containing 5% (w/v) dried milk, with antibodies against hPrp28 (ref. 20), hSnu114 (ref. 51), His_6_-tag (Qiagen, catalogue number 34660), hPrp31 (ref. 35) or phosphorylated hPrp31 (ref. 35), and bound antibody was detected using an ECL detection kit (GE Healthcare). After sequential incubation with His_6_-tag (1:2,000), phosphorylated hPrp31 (1:5,000) and then hSnu114 (1:6,000) antibodies, bound antibodies were removed from the blot by incubating in S-buffer (62.5 mM Tris-HCl pH 6.7, 2% (w/v) SDS, 100 mM β-mercaptoethanol) at 50 °C for 30 min. The blot was then incubated with anti-hPrp28 (1:1,000) followed by anti-hPrp31 (1:1,000) antibodies. See Supplementary Fig. 8 for uncropped western blot images.

### Copurification of 5′ss RNA oligonucleotide with spliceosomes

To determine whether the 5′ss-containing oligonucleotides bind to spliceosomal complexes, unmodified or 2′O-ribose methylated 5′ss RNA oligonucleotide were first radioactively labelled at their 5′-end using γ-^32^P-ATP and T4 polynucleotide kinase. Spliceosomal complexes were allowed to form for 3 min at 30 °C, then a 100-fold molar excess (relative to the MINX-MS2 pre-mRNA) of radiolabelled 5′ss oligonucleotide was added to the reaction. After an additional incubation of 3 min at 30 °C, spliceosomal complexes were purified by 10–30% (v/v) glycerol gradient centrifugation and MS2 affinity-selection. RNA was recovered from the eluates, separated by denaturing PAGE and visualized by autoradiography. The specific activity of the 5′ss oligonucleotides was 30,000 c.p.m. per pmol, whereas that of the MINX-MS2 pre-mRNA was 56,000 c.p.m. per pmol. The relative intensity of the 5′ss oligo and the MINX-MS2 pre-mRNA in lane 2 was quantified using a Phosphorimager and ImageQuantTL (GE Healthcare).

### Data availability

The authors declare that the data supporting the findings of this study are available within the article and its Supplementary Information files.

## Additional information

**How to cite this article:** Boesler, C. *et al*. A spliceosome intermediate with loosely associated tri-snRNP accumulates in the absence of Prp28 ATPase activity. *Nat. Commun.* 7:11997 doi: 10.1038/ncomms11997 (2016).

## Supplementary Material

Supplementary Information Supplementary Figures 1-8 and Supplementary Reference

Supplementary Data 1 Excel Table showing the protein composition of affinity-purified 37S pre-B complexes

## Figures and Tables

**Table 1:** Summary of abundant proteins detected in pre-B complexes by 2D gel electrophoresis.

**Protein name**	**Gene name**	**MW (kDa)**	**gi|number**	***S. cerevisiae*** **gene name**
Sm proteins				
SmB/B'	*SNRPB*	24.6	gi|119631003	SMB1
				
U1 snRNP				
U1-70 K	*SNRNP70*	51.6	gi|29568103	SNP1
U1-A	*SNRPA*	31.3	gi|189053747	MUD1
				
U2 snRNP				
U2A′	*SNRPA1*	28.4	gi|50593002	LEA1
U2B″	*SNRPB2*	25.4	gi|119630691	MSL1
SF3a120	*SF3A1*	88.9	gi|5032087	PRP21
SF3a66	*SF3A2*	49.3	gi|116283242	PRP11
SF3a60	*SF3A3*	58.5	gi|158255798	PRP9
SF3b155	*SF3B1*	145.8	gi|54112117	HSH155
SF3b145	*SF3B2*	100.2	gi|33875399	CUS1
SF3b130	*SF3B3*	135.5	gi|54112121	RSE1
SF3b49	*SF3B4*	44.4	gi|5032069	HSH49
				
U5 snRNP				
hPrp8	*PRPF8*	273.7	gi|91208426	PRP8
hBrr2	*SNRNP200*	244.5	gi|40217847	BRR2
hSnu114	*EFTUD2*	109.4	gi|12803113	SNU114
hPrp6	*PRPF6*	106.9	gi|189067252	PRP6
hPrp28	*DDX23*	95.6	gi|193785886	PRP28
U5-40 K	*SNRNP40*	39.3	gi|115298668	–
				
U4/U6 snRNP				
hPrp3	*PRPF3*	77.6	gi|4758556	PRP3
hPrp31	*PRPF31*	55.4	gi|221136939	PRP31
hPrp4	*PRPF4*	58.4	gi|189053699	PRP4
				
U4/U6.U5 tri-snRNP				
hSnu66	*SART1*	90.2	gi|10863889	SNU66
hSad1	*USP39*	65.4	gi|13926071	SAD1
RBM42	*RBM42*	50.4	gi|12804031	–
				
Miscellaneous				
hPrp4 kinase	*PRPF4B*	117.1	gi|158255924	–
ASR2B	*SRRT*	100	gi|13383501	–
CBP80	*NCBP1*	91.8	gi|4505343	STO1

Proteins from affinity-purified pre-B complexes were analysed by 2D gel electrophoresis (Fig. 2) and individual spots were identified by mass spectrometry. Abundant proteins were determined by visual inspection and are grouped according to their association with snRNPs or stage of recruitment.

## References

[b1] Will C. L. & Lührmann R. Spliceosome structure and function. Cold Spring Harb. Perspect. Biol. 3, doi:10.1101/cshperspect.a003707 (2011). 10.1101/cshperspect.a003707 PMC3119917 21441581

[b2] Robberson B. L., Cote G. J. & Berget S. M. Exon definition may facilitate splice site selection in RNAs with multiple exons. Mol. Cell Biol. 10, 84–94 (1990). 2136768 10.1128/mcb.10.1.84-94.1990 PMC360715

[b3] Wahl M. C., Will C. L. & Lührmann R. The spliceosome: design principles of a dynamic RNP machine. Cell 136, 701–718 (2009). 19239890 10.1016/j.cell.2009.02.009

[b4] Nilsen T. W. RNA Structure and Function 279–307Cold Spring Harbor Laboratory Press (1998).

[b5] Valadkhan S. snRNAs as the catalysts of pre-mRNA splicing. Curr. Opin. Chem. Biol. 9, 603–608 (2005). 16242989 10.1016/j.cbpa.2005.10.008

[b6] Staley J. P. & Guthrie C. Mechanical devices of the spliceosome: motors, clocks, springs, and things. Cell 92, 315–326 (1998). 9476892 10.1016/s0092-8674(00)80925-3

[b7] Liu Y. C. & Cheng S. C. Functional roles of DExD/H-box RNA helicases in Pre-mRNA splicing. J. Biomed. Sci. 22, 54 (2015). 26173448 10.1186/s12929-015-0161-z PMC4503299

[b8] Semlow D. R. & Staley J. P. Staying on message: ensuring fidelity in pre-mRNA splicing. Trends Biochem. Sci. 37, 263–273 (2012). 22564363 10.1016/j.tibs.2012.04.001 PMC3735133

[b9] Cordin O. & Beggs J. D. RNA helicases in splicing. RNA Biol. 10, 83–95 (2013). 23229095 10.4161/rna.22547 PMC3590240

[b10] Teigelkamp S., Mundt C., Achsel T., Will C. L. & Lührmann R. The human U5 snRNP-specific 100-kD protein is an RS domain-containing, putative RNA helicase with significant homology to the yeast splicing factor Prp28p. RNA 3, 1313–1326 (1997). 9409622 PMC1369570

[b11] Strauss E. J. & Guthrie C. PRP28, a 'DEAD-box' protein, is required for the first step of mRNA splicing *in vitro*. Nucleic Acids Res. 22, 3187–3193 (1994). 7520570 10.1093/nar/22.15.3187 PMC310295

[b12] Chang T. H., Latus L. J., Liu Z. & Abbott J. M. Genetic interactions of conserved regions in the DEAD-box protein Prp28p. Nucleic Acids Res. 25, 5033–5040 (1997). 9396812 10.1093/nar/25.24.5033 PMC147153

[b13] Jacewicz A., Schwer B., Smith P. & Shuman S. Crystal structure, mutational analysis and RNA-dependent ATPase activity of the yeast DEAD-box pre-mRNA splicing factor Prp28. Nucleic Acids Res. 42, 12885–12898 (2014). 25303995 10.1093/nar/gku930 PMC4227776

[b14] Laggerbauer B., Achsel T. & Lührmann R. The human U5-200kD DEXH-box protein unwinds U4/U6 RNA duplices in vitro. Proc. Natl Acad. Sci. USA 95, 4188–4192 (1998). 9539711 10.1073/pnas.95.8.4188 PMC22463

[b15] Yang F. . Splicing proofreading at 5' splice sites by ATPase Prp28p. Nucleic Acids Res. 41, 4660–4670 (2013). 23462954 10.1093/nar/gkt149 PMC3632134

[b16] Möhlmann S. . Structural and functional analysis of the human spliceosomal DEAD-box helicase Prp28. Acta Crystallogr. D Biol. Crystallogr. 70, 1622–1630 (2014). 24914973 10.1107/S1399004714006439 PMC4051504

[b17] Staley J. P. & Guthrie C. An RNA switch at the 5' splice site requires ATP and the DEAD box protein Prp28p. Mol. Cell 3, 55–64 (1999). 10024879 10.1016/s1097-2765(00)80174-4

[b18] Chen J. Y. . Specific alterations of U1-C protein or U1 small nuclear RNA can eliminate the requirement of Prp28p, an essential DEAD box splicing factor. Mol. Cell 7, 227–232 (2001). 11172727 10.1016/s1097-2765(01)00170-8

[b19] Hage R. . A targeted bypass screen identifies Ynl187p, Prp42p, Snu71p, and Cbp80p for stable U1 snRNP/Pre-mRNA interaction. Mol. Cell Biol. 29, 3941–3952 (2009). 19451230 10.1128/MCB.00384-09 PMC2704744

[b20] Mathew R. . Phosphorylation of human PRP28 by SRPK2 is required for integration of the U4/U6-U5 tri-snRNP into the spliceosome. Nat. Struct. Mol. Biol. 15, 435–443 (2008). 18425142 10.1038/nsmb.1415

[b21] Roscigno R. F. & Garcia-Blanco M. A. SR proteins escort the U4/U6.U5 tri-snRNP to the spliceosome. RNA 1, 692–706 (1995). 7585254 PMC1369311

[b22] Makarova O. V., Makarov E. M. & Lührmann R. The 65 and 110 kDa SR-related proteins of the U4/U6.U5 tri-snRNP are essential for the assembly of mature spliceosomes. EMBO J. 20, 2553–2563 (2001). 11350945 10.1093/emboj/20.10.2553 PMC125249

[b23] Meister G. . SMNrp is an essential pre-mRNA splicing factor required for the formation of the mature spliceosome. EMBO J. 20, 2304–2314 (2001). 11331595 10.1093/emboj/20.9.2304 PMC125440

[b24] Rappsilber J., Ajuh P., Lamond A. I. & Mann M. SPF30 is an essential human splicing factor required for assembly of the U4/U5/U6 tri-small nuclear ribonucleoprotein into the spliceosome. J. Biol. Chem. 276, 31142–31150 (2001). 11331295 10.1074/jbc.M103620200

[b25] Bessonov S. . Characterization of purified human Bact spliceosomal complexes reveals compositional and morphological changes during spliceosome activation and first step catalysis. RNA 16, 2384–2403 (2010). 20980672 10.1261/rna.2456210 PMC2995400

[b26] Agafonov D. E. . Semiquantitative proteomic analysis of the human spliceosome via a novel two-dimensional gel electrophoresis method. Mol. Cell Biol. 31, 2667–2682 (2011). 21536652 10.1128/MCB.05266-11 PMC3133382

[b27] Schneider M. . Exon definition complexes contain the tri-snRNP and can be directly converted into B-like precatalytic splicing complexes. Mol. Cell 38, 223–235 (2010). 20417601 10.1016/j.molcel.2010.02.027

[b28] Wyatt J. R., Sontheimer E. J. & Steitz J. A. Site-specific cross-linking of mammalian U5 snRNP to the 5' splice site before the first step of pre-mRNA splicing. Genes Dev. 6, 2542–2553 (1992). 1340469 10.1101/gad.6.12b.2542

[b29] Sontheimer E. J. & Steitz J. A. The U5 and U6 small nuclear RNAs as active site components of the spliceosome. Science 262, 1989–1996 (1993). 8266094 10.1126/science.8266094

[b30] Reyes J. L., Kois P., Konforti B. B. & Konarska M. M. The canonical GU dinucleotide at the 5' splice site is recognized by p220 of the U5 snRNP within the spliceosome. RNA 2, 213–225 (1996). 8608445 PMC1369364

[b31] Ismaili N., Sha M., Gustafson E. H. & Konarska M. M. The 100-kda U5 snRNP protein (hPrp28p) contacts the 5' splice site through its ATPase site. RNA 7, 182–193 (2001). 11233976 10.1017/s1355838201001807 PMC1370077

[b32] Cordin O., Banroques J., Tanner N. K. & Linder P. The DEAD-box protein family of RNA helicases. Gene 367, 17–37 (2006). 16337753 10.1016/j.gene.2005.10.019

[b33] Linder P. & Jankowsky E. From unwinding to clamping - the DEAD box RNA helicase family. Nat. Rev. Mol. Cell Biol. 12, 505–516 (2011). 21779027 10.1038/nrm3154

[b34] Price A. M., Gornemann J., Guthrie C. & Brow D. A. An unanticipated early function of DEAD-box ATPase Prp28 during commitment to splicing is modulated by U5 snRNP protein Prp8. RNA 20, 46–60 (2014). 24231520 10.1261/rna.041970.113 PMC3866644

[b35] Schneider M. . Human PRP4 kinase is required for stable tri-snRNP association during spliceosomal B complex formation. Nat. Struct. Mol. Biol. 17, 216–221 (2010). 20118938 10.1038/nsmb.1718

[b36] Anokhina M. . RNA structure analysis of human spliceosomes reveals a compact 3D arrangement of snRNAs at the catalytic core. EMBO J. 32, 2804–2818 (2013). 24002212 10.1038/emboj.2013.198 PMC3817461

[b37] Behzadnia N. . Composition and three-dimensional EM structure of double affinity-purified, human prespliceosomal A complexes. EMBO J. 26, 1737–1748 (2007). 17332742 10.1038/sj.emboj.7601631 PMC1829389

[b38] Boesler C. . Stable tri-snRNP integration is accompanied by a major structural rearrangement of the spliceosome that is dependent on Prp8 interaction with the 5' splice site. RNA 21, 1993–2005 (2015). 26385511 10.1261/rna.053991.115 PMC4604437

[b39] Wolf E. . Exon, intron and splice site locations in the spliceosomal B complex. EMBO J. 28, 2283–2292 (2009). 19536130 10.1038/emboj.2009.171 PMC2726701

[b40] Rigo N., Sun C., Fabrizio P., Kastner B. & Lührmann R. Protein localisation by electron microscopy reveals the architecture of the yeast spliceosomal B complex. EMBO J. 34, 3059–3073 (2015). 26582754 10.15252/embj.201592022 PMC4687791

[b41] Brow D. A. & Guthrie C. Spliceosomal RNA U6 is remarkably conserved from yeast to mammals. Nature 334, 213–218 (1988). 3041282 10.1038/334213a0

[b42] Kuhn A. N., Reichl E. M. & Brow D. A. Distinct domains of splicing factor Prp8 mediate different aspects of spliceosome activation. Proc. Natl Acad. Sci. USA 99, 9145–9149 (2002). 12087126 10.1073/pnas.102304299 PMC123108

[b43] Dignam J. D., Lebovitz R. M. & Roeder R. G. Accurate transcription initiation by RNA polymerase II in a soluble extract from isolated mammalian nuclei. Nucleic Acids Res. 11, 1475–1489 (1983). 6828386 10.1093/nar/11.5.1475 PMC325809

[b44] Das R. & Reed R. Resolution of the mammalian E complex and the ATP-dependent spliceosomal complexes on native agarose mini-gels. RNA 5, 1504–1508 (1999). 10580479 10.1017/s1355838299991501 PMC1369872

[b45] Hartmuth K. . Protein composition of human prespliceosomes isolated by a tobramycin affinity-selection method. Proc. Natl Acad. Sci. USA 99, 16719–16724 (2002). 12477934 10.1073/pnas.262483899 PMC139210

[b46] Kastner B. . GraFix: sample preparation for single-particle electron cryomicroscopy. Nat. Methods 5, 53–55 (2008). 18157137 10.1038/nmeth1139

[b47] Golas M. M., Sander B., Will C. L., Lührmann R. & Stark H. Molecular architecture of the multiprotein splicing factor SF3b. Science 300, 980–984 (2003). 12738865 10.1126/science.1084155

[b48] van Heel M., Harauz G., Orlova E. V., Schmidt R. & Schatz M. A new generation of the IMAGIC image processing system. J. Struct. Biol. 116, 17–24 (1996). 8742718 10.1006/jsbi.1996.0004

[b49] van Heel M. Multivariate statistical classification of noisy images (randomly oriented biological macromolecules). Ultramicroscopy 13, 165–183 (1984). 6382731 10.1016/0304-3991(84)90066-4

[b50] Dube P., Tavares P., Lurz R. & van Heel M. The portal protein of bacteriophage SPP1: a DNA pump with 13-fold symmetry. EMBO J. 12, 1303–1309 (1993). 8467790 10.1002/j.1460-2075.1993.tb05775.x PMC413341

[b51] Fabrizio P., Laggerbauer B., Lauber J., Lane W. S. & Lührmann R. An evolutionarily conserved U5 snRNP-specific protein is a GTP-binding factor closely related to the ribosomal translocase EF-2. EMBO J. 16, 4092–4106 (1997). 9233818 10.1093/emboj/16.13.4092 PMC1170032

[b52] Boehringer D. . Three-dimensional structure of a pre-catalytic human spliceosomal complex B. Nat. Struct. Mol. Biol. 11, 463–468 (2004). 15098019 10.1038/nsmb761

