# Supplementary material for: A spliceosome intermediate with loosely associated tri-snRNP accumulates in the absence of Prp28 ATPase activity
Source: Nat Commun. 2016 Jul 5;7:11997. doi: 10.1038/ncomms11997 (PMC4935976; doi:10.1038/ncomms11997)
Supplement: Supplementary Information — Supplementary Figures 1-8 and Supplementary Reference [file ncomms11997-s1.pdf]

Supplementary Figure 1

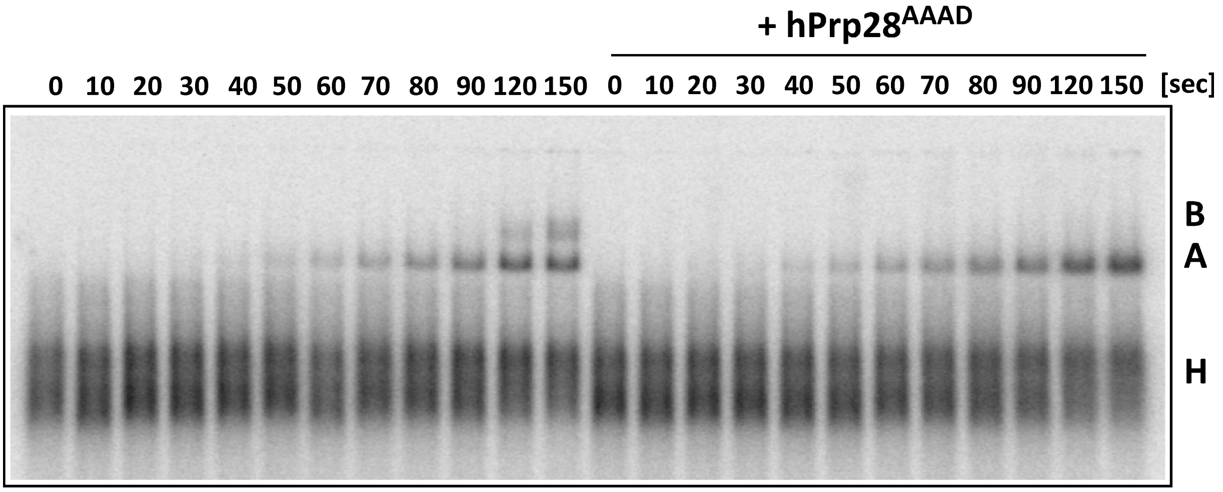

Prp28^{AAAD} does not alter the kinetics of spliceosomal A complex formation. Splicing complexes were assembled on ^{32}P-labelled MINX-MS2 pre-mRNA in HeLa nuclear extract for 0-150 seconds at 30°C, either in the absence or the presence of 50 ng μl^{-1} (final concentration) of recombinant, His_6-tagged hPrp28^{AAAD} protein (as indicated above the lanes), and analysed on a 2% agarose gel in the presence of heparin. The positions of H, A and B complexes are indicated on the right and were visualized with a Phosphorimager.

Supplementary Figure 2

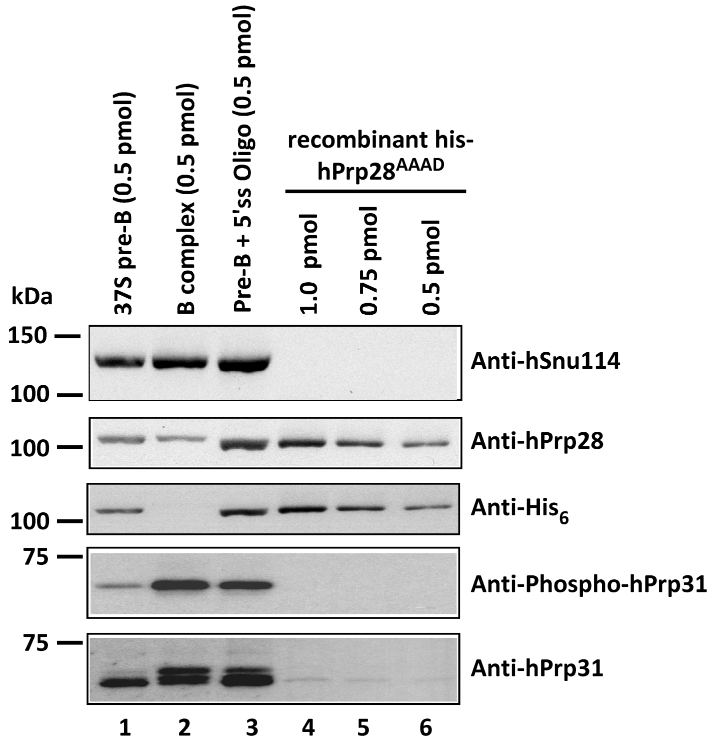

Recombinant hPrp28^{AAAD} protein is incorporated into pre-B complexes and hPrp31 is phosphorylated after pre-B complex formation. Splicing complexes were assembled on MINX-MS2 pre-mRNA in HeLa nuclear extract for 6 min either in the absence (B complex) or the presence of 50 ng μl^{-1} of the recombinant, His_6-tagged hPrp28^{AAAD} protein (37S pre-B complex). Alternatively, pre-B complexes were allowed to form in Hela nuclear extract for 4 min at 30°C and a 100-fold molar excess of the 5'ss RNA oligonucleotide was added to the reaction which was incubated for an additional 4 min. Spliceosomal complexes were fractionated on a 10-30 % (v/v) glycerol gradient containing 150 mM KCl and complexes in 37S (pre-B) or 45S peak fractions were subjected to MS2 affinity-selection. Proteins from the purified complexes (0.5 pmoles) or 0.5, 0.75 or 1.0 pmole of his-tagged hPrp28^{AAAD} were analysed by Western blot using antibodies against hPrp28, His_6-tag, hPrp31 (recognizing both phosphorylated and non-phosphorylated forms of the protein) or specific for phosphorylated hPrp31. Antibodies against hSnu114 were used to ensure equal loading. The upper band detected with anti-hPrp31 antibodies is the phosphorylated form of hPrp31.

Supplementary Figure 3

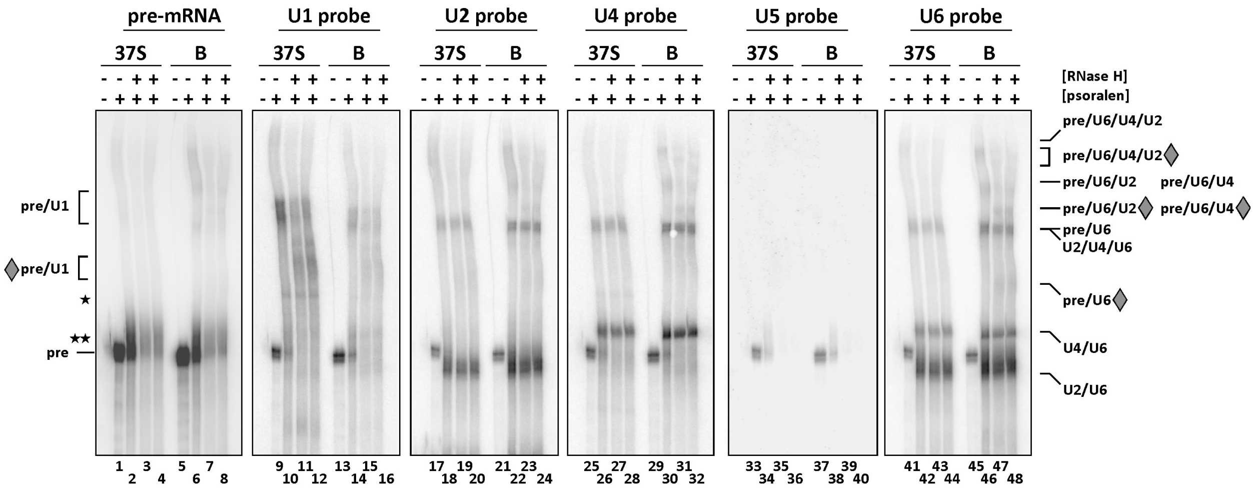

Identification of RNA-RNA interactions in the 37S pre-B complex via psoralen crosslinking (shorter exposure). Affinity-purified 37S pre-B and B complexes formed on ^{32}P-labelled MINX-MS2 pre-mRNA were UV-irradiated +/- psoralen (AMT) and total psoralen-crosslinked RNA was incubated with RNase H and an oligonucleotide complementary to exon 2 of the MINX pre-mRNA as indicated above each lane. RNA-RNA crosslinks were identified by Northern blot analyses, incubating sequentially with ^{32}P-labeled probes against the pre-mRNA, and U1, U2, U4, U5 and U6 snRNAs as described in the legend to Fig. 3. The positions of crosslinked RNA species are indicated. Bands appearing after RNase H digestion are indicated with a diamond (◊).*, potential U1/U4 crosslink. **, internally crosslinked MINX pre-mRNA.

Supplementary Figure 4

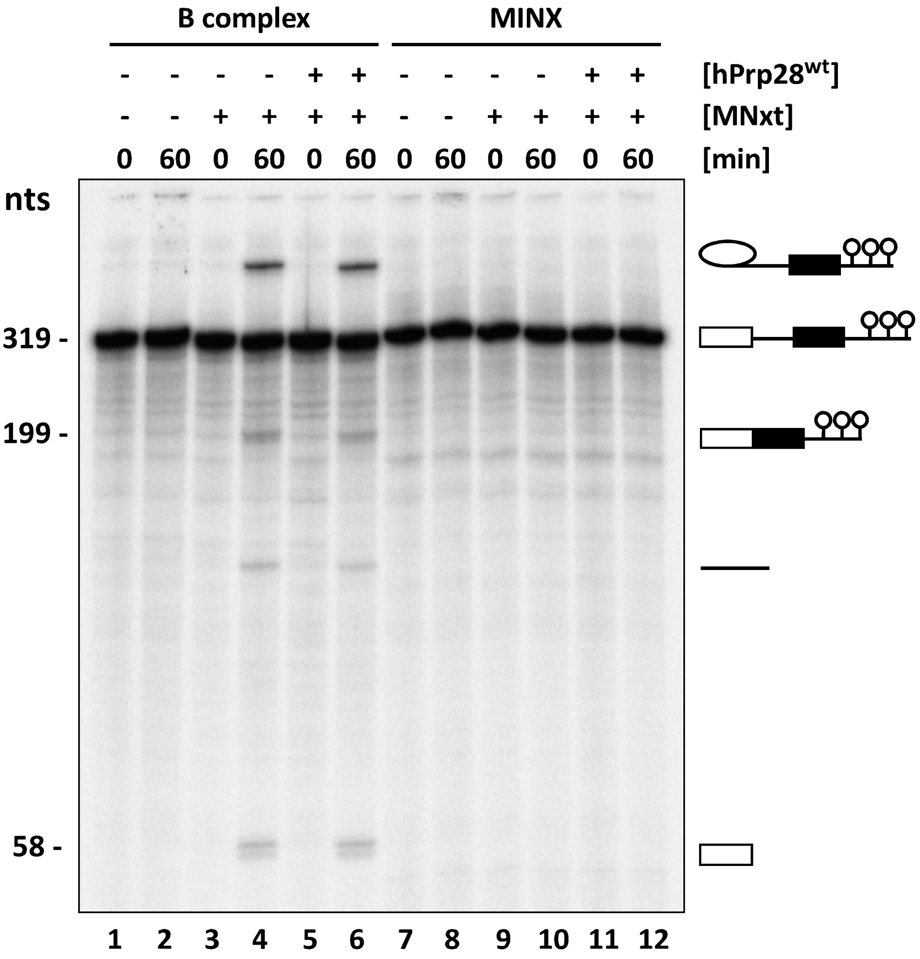

Addition of hPrp28^{wt} protein does not enhance the efficiency of the chase of the B complex into a catalytically-active complex. Purified B complexes formed on ^{32}P-labelled MINX-MS2 pre-mRNA (lanes 1- 6) or MINX-MS2 pre-mRNA alone (lanes 7-12) were incubated under splicing conditions in the presence of buffer (lanes 1-2 and 7-8) or micrococcal nuclease-treated HeLa nuclear extract (MNxt) (lanes 3-6 and 9-12). Recombinant hPrp28^{wt} protein was added to a final concentration of 50 μg ml^{-1} as indicated and reactions were incubated on ice for 30 min prior to incubation at 30°C for the indicated times. RNA was analysed by denaturing PAGE and visualized with a Phosphorimager. The positions of the pre-mRNA, splicing intermediates and products are indicated on the right. The size (nucleotide length, nts) of the pre-mRNA, mRNA and 5’ exon is indicated on the left.

Supplementary Figure 5

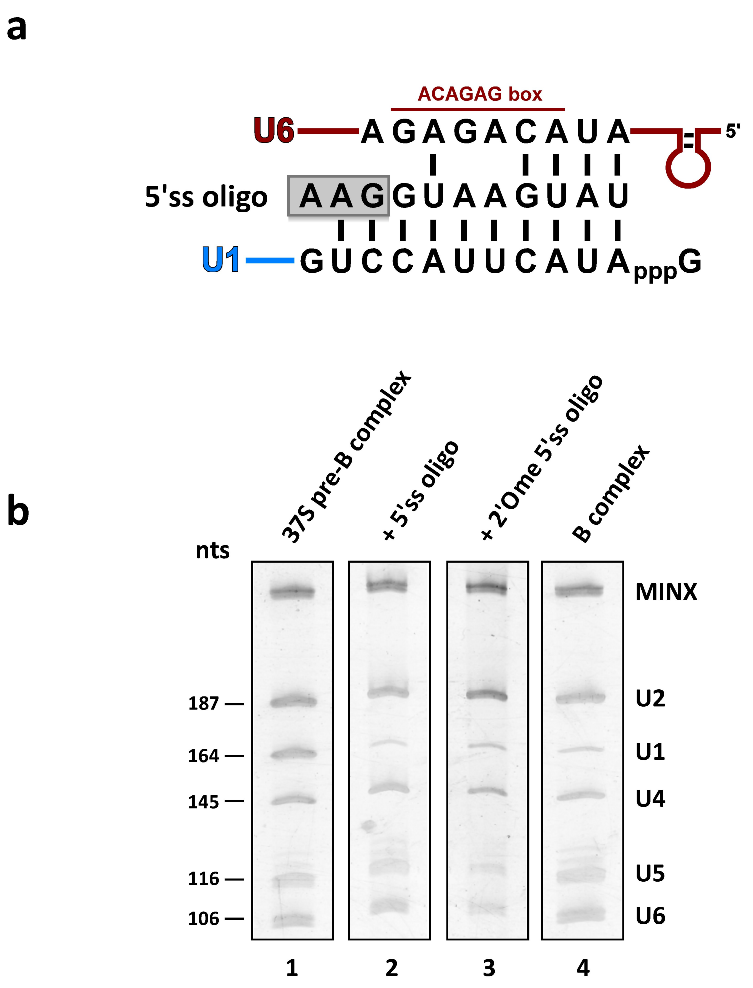

An excess of a 5'ss-containing RNA oligonucleotide leads to a reduction in U1 in affinity-purified spliceosomal complexes. (a) Sequence of the 5'ss oligo and complementary to U1 and U6 snRNA. **(b)** The 5'ss oligo displaces U1 from the 37S pre-B complex. 37S cross-intron complexes were allowed to form in splicing extract in the presence of an inhibitory concentration of hPrp28^{AAAD} for 3 min at 30°C. Subsequently, a 100-fold excess of wildtype 5'ss oligonucleotide or a 2'Ome version thereof was added and the reaction incubated for an additional 3 min. Reactions were subjected to glycerol gradient centrifugation in the presence of 150 mM KCl. B complexes (6 min splicing reaction) were run in parallel. After fractionation of the gradient, complexes in the ~37S or 45S peak fractions were affinity-purified, and their RNA was analysed by denaturing PAGE and visualized by silver staining. Identities of the snRNAs are indicated on the right

Supplementary Figure 6

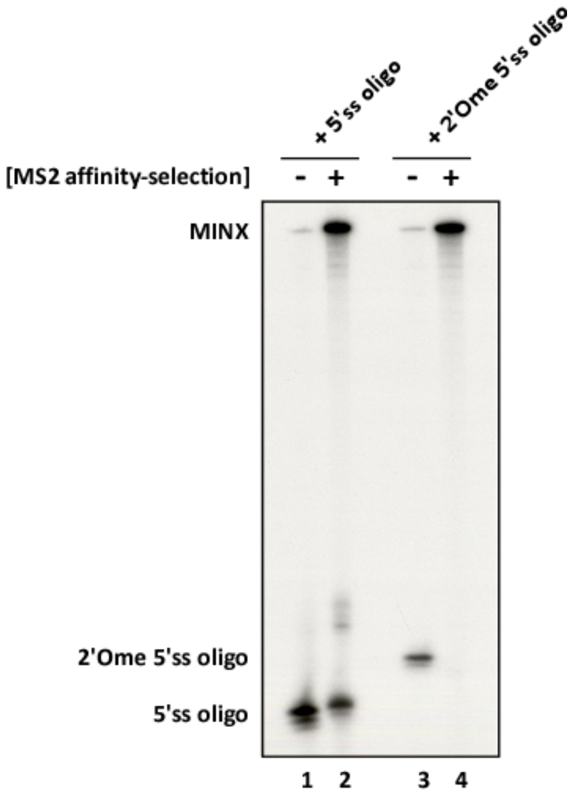

The 5'ss oligo, but not a 2'Ome modified version of it, is stably-associated with the 37S pre-B complex. 37S pre-B complexes were assembled in splicing reactions for 3 min at 30°C, then a 100-fold excess (relative to the pre-mRNA) of an unmodified or 2'O-ribose methylated (2'Ome), 5'-^{32}P-labeled 5'ss RNA oligonucleotide was added to the splicing reaction. Lanes 1 and 3, Input (1% of the total reaction) after incubation in the splicing reactions. Subsequently, the reactions were subjected to glycerol gradient centrifugation and spliceosomal complexes were purified by MS2 affinity-selection from the respective peak fractions. RNA was recovered, separated by denaturing PAGE and visualized by autoradiography. The positions of MINX-MS2 pre-mRNA and the 5'-labeled oligonucleotides are indicated. Due to the large excess of the 5'ss oligos relative to the MINX-MS2 RNA, the signal of the MINX-MS2 pre-mRNA is much lower in lanes 1 and 3. The specific activity of the 5'ss oligonucleotides was 30,000 cpm per pmole, whereas that of the MINX-MS2 pre-mRNA was 56,000 cpm per pmole. The relative intensity of the 5'ss oligo and MINX-MS2 pre-mRNA in lane 2 was quantitated, revealing a 0.87:1 (oligo versus pre-mRNA) stoichiometry in the pre-B complex.

Supplementary Figure 7

a

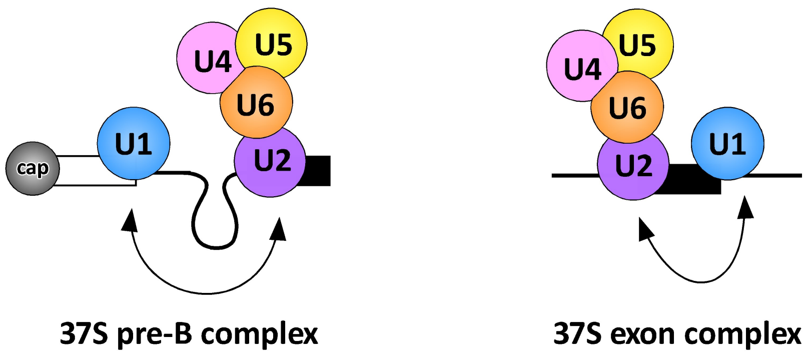

b

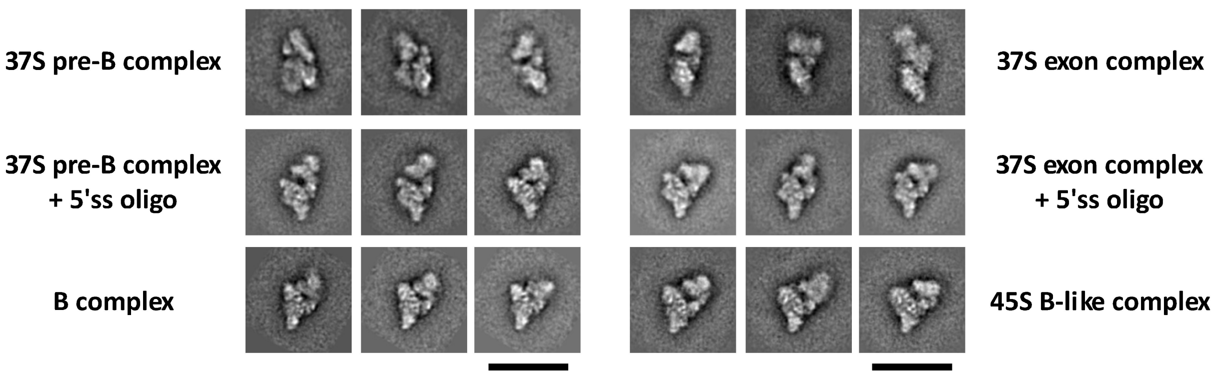

Comparison of the morphology of the 37S cross exon and 37S pre-B complexes. (**a**) Schematic of the likely orientation of the U1, U2, U4, U5 and U6 snRNPs in the 37S pre-B and cross exon complex. (**b**) Complexes were affinity purified and visualized by negative-stain electron microscopy after gradient fixation (GraFix). The most abundant class averages of the indicated spliceosomal complexes are shown. Bar = 50 nm. Class averages of the 37S exon complex (plus and minus 5'ss oligo) and 45S B-like complexes are from Boesler et al., 2015(ref 1).

Supplementary Figure 8

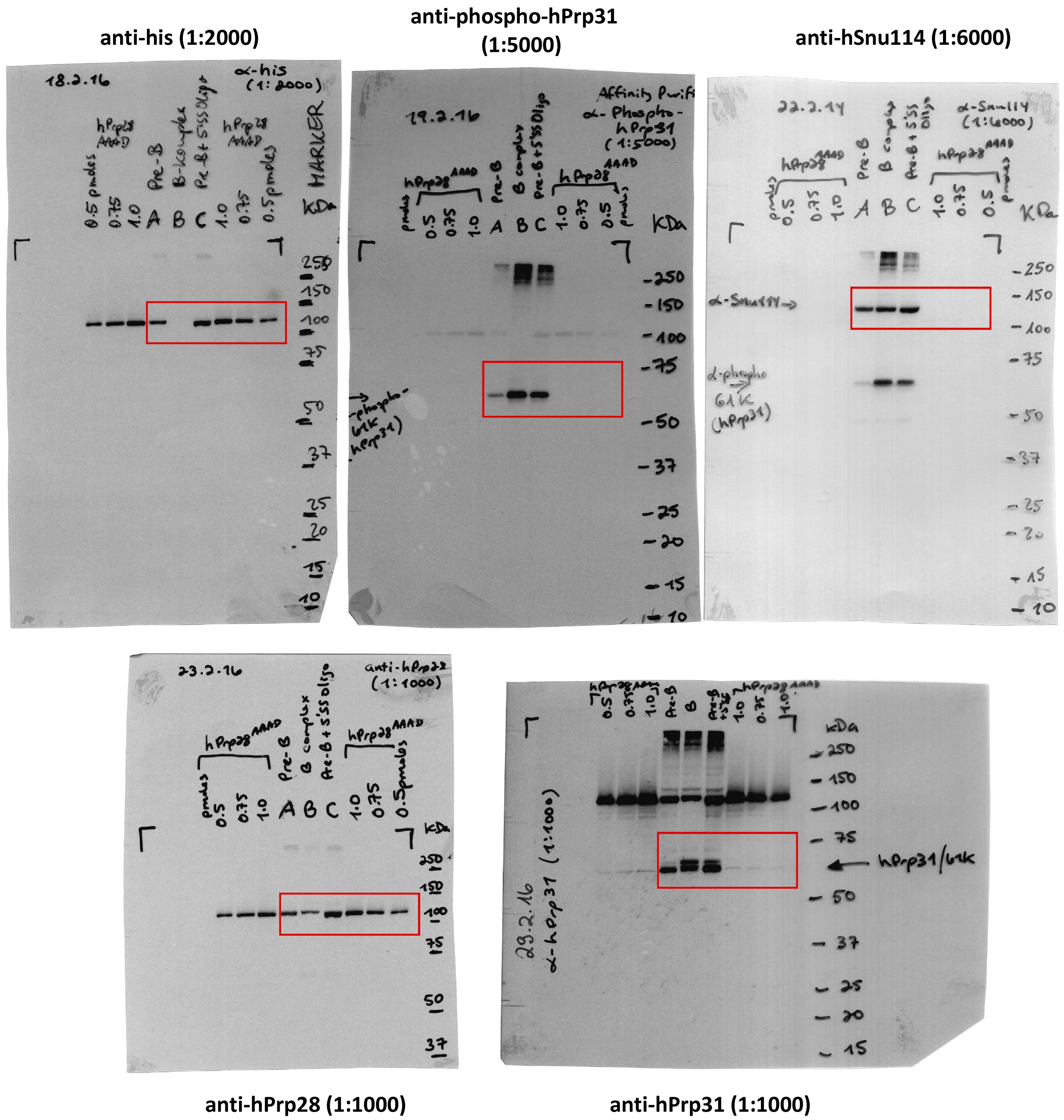

Uncropped Western blots related to Supplementary Figure 2. The membrane was first incubated with anti-his antibodies followed by anti-phospho-hPrp31 and then anti-hSnu114 antibodies. Bound antibodies were stripped from the blot and subsequently it was incubated with anti-hPrp28 and anti-hPrp31 antibodies. The antibody dilutions used are indicated.

Supplementary References

1. Boesler, C. *et al.* Stable tri-snRNP integration is accompanied by a major structural rearrangement of the spliceosome that is dependent on Prp8 interaction with the 5' splice site. *RNA* **21**, 1993-2005 (2015).
